# Combined toxicity of pesticides and heavy metals on *Rhizoglyphus robini* (Acari: Acaridae)

**DOI:** 10.1038/s41598-026-59265-5

**Published:** 2026-06-22

**Authors:** Hosein Samadieh, Jahangir Khajehali

**Affiliations:** Department of Plant Protection, College of Agriculture, Isfahan University of Technology, Isfahan, 84156-83111 Iran

**Keywords:** Bulb mite, Pesticides, Detoxification enzymes, Synergistic, Nickel, Environmental sciences, Risk factors, Zoology

## Abstract

This study investigates the combined effects of heavy metals (lead and nickel) and three pesticides (abamectin, propargite, and chlorpyrifos) on the bulb mite, Rhizoglyphus robini (Acaridae). Toxicity bioassays were conducted to determine the heavy metal ratios (HMR) and the LC_50_ of pesticide alone divided by LC_50_ of pesticide with heavy metal pretreatment. The results revealed that exposure to lead and nickel enhanced the toxicity of propargite and chlorpyrifos but significantly reduced the toxicity of abamectin, indicating an antagonistic effect. Specifically, for propargite, lead increased toxicity by 2.67-fold and nickel by 1.73-fold. For chlorpyrifos, lead increased toxicity by 1.75-fold and nickel by 1.17-fold. In contrast, for abamectin, lead and nickel reduced toxicity by 0.82-fold and 0.86-fold, respectively (HMR < 1), confirming an antagonistic interaction. Biochemical analyses showed that lead exposure significantly increased cytochrome P450 activity, while nickel reduced glutathione S-transferase (GST) activity. The observed changes in enzyme activities suggest that heavy metals may affect detoxification pathways. Our results highlight the importance of considering environmental contaminants when designing pest management strategies, as combined exposure to heavy metals and pesticides can either enhance or reduce the efficacy of control measures.

**Supplementary Information:**

The online version contains supplementary material available at https://doi.org/10.1038/s41598-026-59265-5.

## Introduction

Saffron (*Crocus sativus* L.) is one of the most economically important spice and medicinal crops worldwide. Its corms are highly susceptible to infestation by the bulb mite, *Rhizoglyphus robini*, which can cause substantial yield and quality losses during cultivation and storage^1^.

Mites of the family Acaridae are major pests in agriculture and stored products. Bulb mites of the genus *Rhizoglyphus*, particularly *R. echinopus* and *R. robini*, are globally distributed and damage plants growing from bulbs, corms, and tubers^2^. In Iran, *R. robini* is a key pest of saffron, as it infests corms, feeding on their outer layers and causing reduced vigor, stunted growth, and potential crop loss^3^. These mites thrive in moist environments rich in organic matter, such as saffron fields, and infestations during storage further compromise planting material and yields^4^. Effective management includes maintaining optimal soil moisture, using resistant saffron varieties, and applying biological or chemical controls. Developing integrated pest management strategies for *R. robini* is essential for sustainable saffron production in Iran^5^.

Organisms in natural ecosystems are continuously exposed to complex mixtures of xenobiotics, including heavy metals, pesticides, and toxic gases. These compounds can induce toxicity in both animals and humans by disrupting key physiological systems and vital organs^6^. Mixtures of pesticides and heavy metals, such as dimethoate with cadmium or lead, have also been associated with immunotoxicity, including changes in body weight, organ integrity, and immune function^7^. Given the ubiquitous presence of xenobiotics in the environment, understanding their interactive and cumulative biological effects is essential for accurate risk assessment.

Over the recent decades, the anthropogenic enrichment of heavy metals in agricultural soils, predominantly mediated via industrial waste, agrochemical inputs, and atmospheric deposition, has posed a severe threat to sustainable agriculture^8^. Heavy metals, most notably lead (Pb), cadmium (Cd), nickel (Ni), and zinc (Zn), exhibit extreme persistence and recalcitrance against degradation, leading to progressive bioaccumulation within the soil profile^9^. This accumulation substantially degrades soil health by shifting microbial community dynamics, disrupting nutrient cycling, and compromising the overall physicochemical properties of the edaphic environment^10^. Within heavily managed agroecosystems, the ecotoxicological ramifications of heavy metal pollution extend beyond plant phytotoxicity to influence pest management dynamics^11^. Root and corm crops, such as saffron, are critically vulnerable to the subterranean absorption of these contaminants, which threatens both crop physiology and food safety^12^. Crucially, the intersection of heavy metal stress and pesticide application creates a multi-stress environment that can drive synergistic or antagonistic outcomes, altering conventional pest suppression efficacy^6^. Emerging evidence suggests that heavy metals act as potent modulators of pest counter-defenses, particularly affecting the enzymatic detoxification cascades in major pests like mites^13^. Intriguingly, this joint action often manifests as a synergistic effect; exposure to metals like Pb and Ni can suppress the pest’s metabolic detoxification machinery, unexpectedly accelerating their susceptibility to chemical treatments^14^.

Heavy metal pollution has emerged as a critical environmental stressor that not only exerts ecotoxicological effects along the food chain but also complicates pest management strategies. Recent research highlights that prior exposure to heavy metals can induce cross-tolerance in pests to subsequent insecticides by priming metabolic enzymes. This phenomenon introduces unprecedented challenges to integrated pest management (IPM) in heavy metal-contaminated areas^12^.

Despite extensive knowledge of pesticide toxicity, information on the combined effects of heavy metals and pesticides on *R. robini* is limited. Understanding these interactions is critical for predicting the effectiveness of chemical control under field conditions, particularly in saffron fields where heavy metal contamination is common. Beyond canonical genetic mechanisms, recent insights into environmental-chemical interactions highlight a novel dimension of resistance. Heavy metal pollution synergistically accelerates insecticide resistance; as primary stressors, these metals hyper-activate physiological stress pathways and detoxifying enzymes (cytochrome P450s and GSTs). This priming effect exponentially enhances the insect’s biochemical detoxification capacity upon subsequent insecticide exposure, severely compromising chemical control efficacy^15^.

*R. robini* is an economically important pest of saffron corms in Iran, and chemical control remains one of the principal management approaches against this species. Since saffron is cultivated in soils that may contain elevated levels of heavy metals, understanding how metal exposure influences pesticide susceptibility is important for predicting control efficacy under field conditions.

In this study, we investigated the combined effects of three pesticides (abamectin, propargite, and chlorpyrifos) and four heavy metals (Pb, Ni, Cd, and Zn) on *R. robini*. Additionally, the activities of detoxification enzymes, including cytochrome P450s, GSTs, and esterases, were measured to elucidate the physiological mechanisms underlying changes in pesticide susceptibility following heavy metal exposure.

## Materials and methods

### Mite’s population and rearing

Saffron corms were collected from fields in Neyshabur City, Razavi Khorasan province, where visible signs of mite infestation were observed, and transported to the laboratory for detailed analysis. In the laboratory, the samples were initially placed in a germinator for one week to facilitate the rapid growth of the mite population. Following this period, adults and immature stages of mites collected from the infested corms were transferred into 9 cm Petri dishes lined with two layers of filter paper soaked in distilled water at 25 ± 1 °C and a relative humidity of % 60. The mites were provided with raw peanut kernels as a food source^16^. These Petri dishes were positioned within 25 × 15 cm plastic boxes, which were then placed inside a growth chamber in total darkness. Peanuts and distilled water were replenished every three days to sustain optimal humidity levels.

### Test chemicals and heavy metals

Commercial formulations of abamectin (Golsam), propargite (Paksam), and chlorpyrifos (Golsam) were used in toxicity bioassays. The heavy metals used were nickel (Ni), lead (Pb), cadmium (Cd), and zinc (Zn), all prepared as nitrate solutions in 0.5 mol/L HNO₃, except for cadmium, which was in 0.5 mol/L HNO₃. Each solution contained 1000 mg a.i./L of the respective metal, provided as Certipur^®^ standards from Sigma-Aldrich. Each bioassay included a control group along with four different pesticide concentrations. The concentrations were carefully prepared based on results from preliminary tests. A 5 cm diameter filter paper disc (Whatman No. 1) was placed at the bottom of a 5 cm diameter Petri dish, with 1.5 ml of the chemical solution applied. Preliminary bioassays were conducted to determine suitable concentration ranges producing 10–90% mortality. Four concentrations were tested for each pesticide. Each concentration was replicated four times using 20–30 adult female mites per replicate. Mortality was recorded after 48 h and corrected using Abbott’s formula when necessary. LC_50_ values and their 95% confidence intervals were estimated using probit analysis in PoloPlus software. In the bioassay conducted to assess the impact of heavy metals, a group of 200 female adult mites was exposed to a concentration of 100 mg a.i./L for each heavy metal over 72 h. After this exposure period, the mites were transferred to a growth environment to confirm that heavy metal treatment did not affect their fitness. Following this exposure, the mites were used to evaluate the toxicity of pesticides in the bioassay, with distilled water serving as the control in this experiment. The bioassay of these mites was carried out similarly to the bioassay of pesticides, and the median lethal concentration for each compound was determined. In measuring the activity of detoxifying enzymes, these mites were also exposed to a concentration of 100 mg a.i./L of heavy metals for 72 h. The concentration of 100 mg a.i./L was selected based on preliminary laboratory experiments and supported by previous acarological studies demonstrating that heavy metal exposures around 100 mg/kg are sufficient to induce measurable physiological and detoxification responses in mites without causing complete mortality. Previous studies on *Aleuroglyphus ovatus* reported significant alterations in antioxidant and detoxification systems following exposure to 100 mg/kg lead, supporting the use of comparable exposure levels for mechanistic investigations^17^. Based on the preliminary mortality bioassays, Pb and Ni were selected for subsequent biochemical analyses because they produced the strongest effects on pesticide toxicity compared with Cd and Zn. Since the objective of the enzyme assays was to investigate the physiological mechanisms underlying the most pronounced heavy metal–pesticide interactions, detailed analyses were focused on Pb and Ni. Cadmium and zinc were therefore included only in the initial toxicity screening to provide a broader comparison among commonly occurring heavy metals. Each treatment in both the mortality and enzyme activity bioassays was performed with four independent biological replicates. The Heavy Metal Ratio (HMR) was calculated to evaluate the interactive effects of heavy metals on pesticide toxicity using the following equation:

$$\:\mathrm{H}\mathrm{M}\mathrm{R}=\:\frac{\mathrm{L}\mathrm{C}50\:\mathrm{o}\mathrm{f}\:\mathrm{p}\mathrm{e}\mathrm{s}\mathrm{t}\mathrm{i}\mathrm{c}\mathrm{i}\mathrm{d}\mathrm{e}\:\mathrm{a}\mathrm{l}\mathrm{o}\mathrm{n}\mathrm{e}}{\mathrm{L}\mathrm{C}50\mathrm{o}\mathrm{f}\:\mathrm{p}\mathrm{e}\mathrm{s}\mathrm{t}\mathrm{i}\mathrm{c}\mathrm{i}\mathrm{d}\mathrm{e}\:\mathrm{w}\mathrm{i}\mathrm{t}\mathrm{h}\:\mathrm{h}\mathrm{e}\mathrm{a}\mathrm{v}\mathrm{y}\:\mathrm{m}\mathrm{e}\mathrm{t}\mathrm{a}\mathrm{l}\:\mathrm{p}\mathrm{r}\mathrm{e}\mathrm{t}\mathrm{r}\mathrm{e}\mathrm{a}\mathrm{t}\mathrm{m}\mathrm{e}\mathrm{n}\mathrm{t}}$$

### The approach for preparing enzyme samples and assessing their activity

To measure CYP450 activity, 100 mites were first homogenized on ice in 500 µL of potassium phosphate buffer (0.1 M, pH 7). The homogenate was centrifuged at g × 10,000 for 20 min at 4 °C, and the resulting supernatant was used as the enzyme source. Following standard procedures, CYP450 activity was assessed using 3,3’,5,5’-Tetramethylbenzidine (TMBZ) as a substrate. The reaction mixture contained 400 µL of TMBZ, 50 µL of 3% hydrogen peroxide, 160 µL of potassium phosphate buffer (0.625 M, pH 7.2), and 40 µL of the enzyme extract. The mixture was incubated at room temperature in the dark for 2 h, after which absorbance was measured at 450 nm with a spectrophotometer (UNICO, Dayton, USA). The CYP450 activity was expressed as units per milligram of protein^18^. For esterase (EST) activity, 100 mites were homogenized on ice in 500 µL of 0.1 M sodium phosphate buffer (pH 7.5) containing 0.1% (w/v) Triton X-100. The homogenate was centrifuged at g ×10,000 for 15 min at four °C, and the supernatant was retained for further analysis. EST activity was determined using a modified version of Grant’s method^19^. The substrate solution dissolved 15 mg of Fast Blue RR salt in 25 mL of 0.2 M sodium phosphate buffer (pH 6.0) and added 250 µL of 100 mM α-Naphthyl Acetate (α-NA) in acetone. The final reaction mixture, with a volume of 500 µL, consisted of 50 µL sodium phosphate buffer, 400 µL substrate solution, and 50 µL enzyme extract. Absorbance was recorded at 450 nm at 30-second intervals over 5 min using a spectrophotometer (UNICO, Dayton, USA). EST activity was expressed as nmol of α-naphthol produced per milligram of protein per minute. Glutathione S-transferase (GST) activity was measured by homogenizing 100 adult mites on ice in 500 µL of sodium phosphate buffer (0.2 M, pH 7). The supernatant was used as the enzyme source after centrifuging the homogenate at g ×12,000 for 15 min at 4 °C. GST activity was measured using 1-Chloro-2,4-Dinitrobenzene (CDNB) as the substrate and reduced Glutathione (GSH). The reaction mixture, totaling 430 µL, included 200 µL of CDNB, 200 µL of GSH, and 30 µL of the supernatant. Absorbance was monitored at 340 nm every 30 s for 5 min using a spectrophotometer (UNICO, Dayton, USA). GST activity was calculated using an extinction coefficient of 9.6 mM cm ^–1^ and reported as nmol of CDNB conjugated per milligram of protein per minute^18^.

### Statistical analysis

Statistical analyses were performed using Polo-Plus 2.0 software to calculate the LC_50_ values^20^. LC_50_ values and their 95% confidence intervals were estimated by probit analysis. Enzyme activity data were analyzed by one-way analysis of variance (ANOVA), and treatment means were separated using Tukey’s HSD test at *P* ≤ 0.05. Data are presented as mean ± standard error (SE). Data visualization was performed using R software (R Foundation for Statistical Computing, Vienna, Austria).

## Results

### Acute toxicity of the median lethal concentration of pesticides with and without pretreatment of heavy metals

**Fig. 1:**
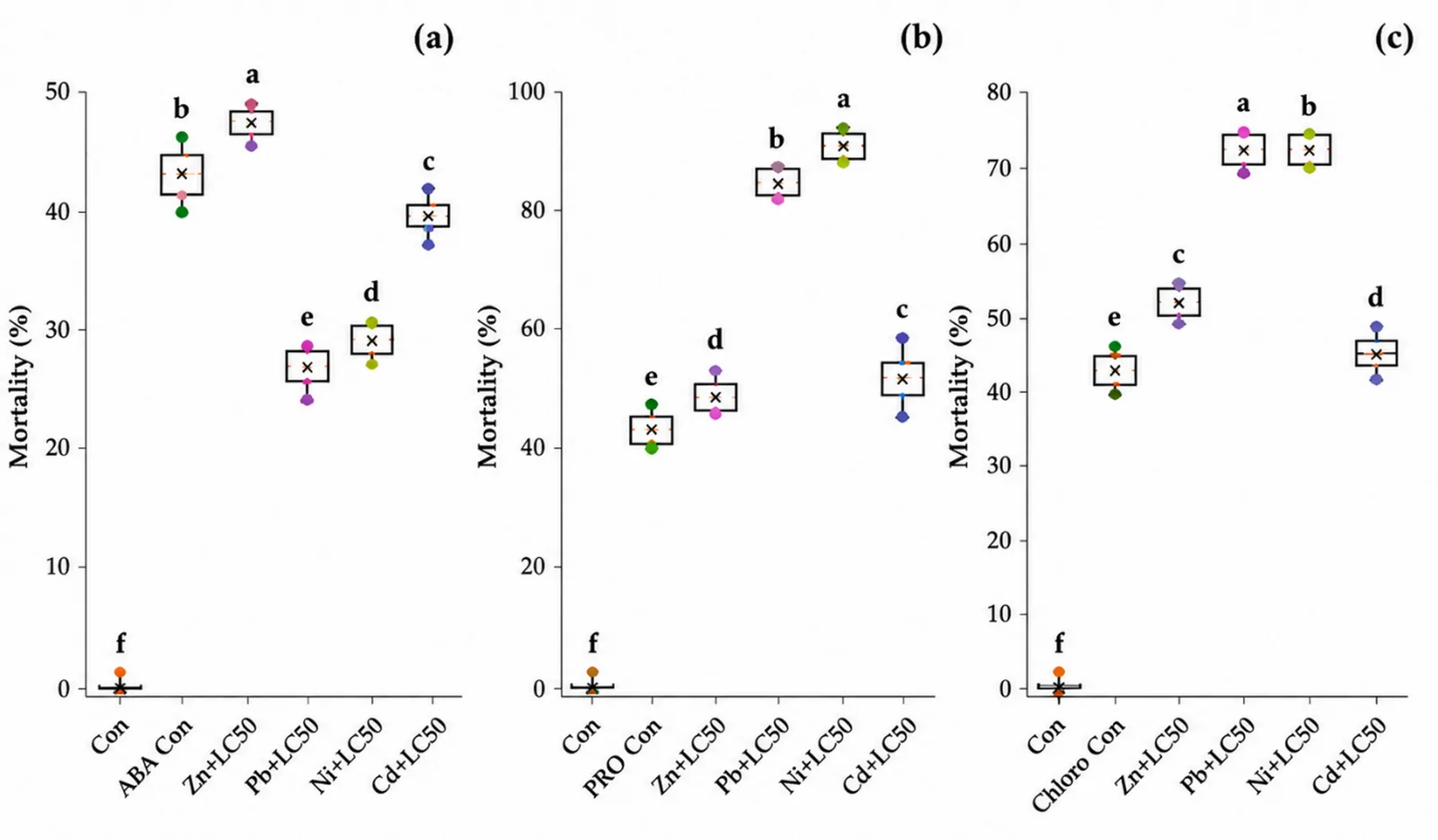
Mortality (%) of *R. robini* adults in control (distilled water alone), Pesticide-only (LC_50_), and Pesticide (LC_50_) treatments after a 72-h pretreatment with a fixed concentration (100 mg a.i./L) of selected heavy metals: (**a**) Abamectin, (**b**) Propargite, and (**c**) Chlorpyrifos. Boxes represent the interquartile range (IQR) with median (horizontal line); whiskers indicate the minimum and maximum values. Points represent the four replicates (*n* = 4) and “×” denotes the mean. Different letters above the boxes indicate significant differences among treatments within each Pesticide based on Tukey’s HSD test (*P* < 0.05).

The mortality of *R. robini* adults varied considerably among insecticide treatments and heavy metal pretreatments (Fig. 1). In the abamectin assay, no mortality was observed in the untreated control group, whereas exposure to abamectin (2.5 mg a.i./L) alone resulted in approximately 46% mortality. Pretreatment with Zn increased mortality slightly compared with abamectin alone, while Pb and Ni pretreatments were associated with lower mortality levels. Cadmium pretreatment produced mortality values similar to those observed in the abamectin-only treatment.

In the propargite assay, mortality in the untreated control remained at 0%, whereas propargite (30 mg a.i./L) alone caused approximately 44% mortality. The highest mortality levels were recorded in mites pretreated with Pb and Ni, reaching approximately 88% and 92%, respectively. Zinc and cadmium pretreatments also increased mortality relative to propargite alone, although their effects were less pronounced than those of Pb and Ni.

For chlorpyrifos, mortality in the insecticide-only treatment (0.8 mg a.i./L) was approximately 45%. Zinc pretreatment increased mortality to about 54%, whereas Pb and Ni pretreatments resulted in substantially higher mortality levels, exceeding 70%. Cadmium pretreatment produced mortality values comparable to those observed in the chlorpyrifos-only treatment.

Overall, the effect of heavy metal pretreatment depended on both the metal and the insecticide used. Pb and Ni pretreatments generally enhanced the toxicity of propargite and chlorpyrifos, whereas their effects on abamectin were less pronounced. These findings suggest that prior exposure to certain heavy metals may alter the susceptibility of *R. robini* to insecticides.

Based on the mortality bioassays, Pb and Ni showed the most pronounced effects on the toxicity of the tested pesticides compared with Zn and Cd. Since these metals produced the greatest changes in mortality responses, they were selected for subsequent enzymatic assays to provide further insight into the biochemical basis of their effects on pesticide susceptibility in *R. robini*.

### Acute toxicity of pesticides with and without pretreatment of selected heavy metals

Table 1 presents the heavy metal ratios (HMR) for the three pesticides after pretreatment with lead and nickel. For abamectin, pretreatment with lead and nickel resulted in HMR values of 0.82 and 0.86, respectively, both below 1. This indicates an antagonistic effect: the presence of heavy metals reduced the toxicity of abamectin by approximately 0.82‑fold and 0.86‑fold (the pesticide became less effective).

In contrast, propargite showed a strong synergistic effect. Lead pretreatment increased the HMR by 2.67‑fold, and nickel by 1.73‑fold, meaning that the toxicity of propargite was markedly enhanced in the presence of these heavy metals. Chlorpyrifos also exhibited synergism, with HMR increases of 1.75‑fold (lead) and 1.17‑fold (nickel). Although the effect of nickel was modest, both metals clearly increased the toxicity of chlorpyrifos, though to a lesser extent than observed for propargite.

**Table 1:** Acute toxicity of pesticides (the median lethal concentration) with and without pretreatment of lead and nickel.

Treatment	n^a^	LC_50_ (mg / L) [95% CI]		Slope ± SE	χ2(df)	HMR^b^ (95% CI)
Abamectin	486	2.57 [2.35–2.79]		3.46 ± 0.345	1.28 (14)	-
+ Lead	388	3.13 [2.83–3.44]		3.23 ± 0.376	3.77 (14)	0.82 (0.72–0.93)
+ Nickel	486	2.97 [2.66–3.28]		3.01 ± 0.354	3.89 (14)	0.86 (0.75–0.98)
Propargite	441	30.72 [26.50-35.47]		2.06 ± 0.201	0.54 (14)	-
+ Lead	442	11.50 [10.11–13.04]		2.44 ± 0.215	3.05 (14)	2.67 (2.19–3.24)
+ Nickel	438	17.73 [15.50–20.20]		2.30 ± 0.211	1.52 (14)	1.73 (1.42–2.11)
Chlorpyrifos	481	0.83 [0.77–0.89]		4.16 ± 0.348	6.28 (14)	-
+ Lead	415	0.47 [0.42–0.52]		3.31 ± 0.329	5.70 (14)	1.75 (1.54–1.98)
+ Nickel	428	0.70 [0.64–0.76]		3.83 ± 0.369	4.51 (14)	1.17 (1.05–1.31)

n^a^ all mites tested in bioassay including control.

HRM^b^ $$\left(\mathrm{H}\mathrm{e}\mathrm{a}\mathrm{v}\mathrm{y}\:\mathrm{M}\mathrm{e}\mathrm{t}\mathrm{a}\mathrm{l}\:\mathrm{R}\mathrm{a}\mathrm{t}\mathrm{i}\mathrm{o}\right)\:=\:\frac{\mathrm{L}\mathrm{C}50\:\mathrm{o}\mathrm{f}\:\mathrm{p}\mathrm{e}\mathrm{s}\mathrm{t}\mathrm{i}\mathrm{c}\mathrm{i}\mathrm{d}\mathrm{e}\:\mathrm{a}\mathrm{l}\mathrm{o}\mathrm{n}\mathrm{e}}{\mathrm{L}\mathrm{C}50\mathrm{o}\mathrm{f}\:\mathrm{p}\mathrm{e}\mathrm{s}\mathrm{t}\mathrm{i}\mathrm{c}\mathrm{i}\mathrm{d}\mathrm{e}\:\mathrm{w}\mathrm{i}\mathrm{t}\mathrm{h}\:\mathrm{h}\mathrm{e}\mathrm{a}\mathrm{v}\mathrm{y}\:\mathrm{m}\mathrm{e}\mathrm{t}\mathrm{a}\mathrm{l}\:\mathrm{p}\mathrm{r}\mathrm{e}\mathrm{t}\mathrm{r}\mathrm{e}\mathrm{a}\mathrm{t}\mathrm{m}\mathrm{e}\mathrm{n}\mathrm{t}}$$

### The comparison of detoxification enzyme activity with and without pretreatment of selected heavy metals

The activities of glutathione S-transferase (GST), cytochrome P450 (P450), and esterase (EST) were significantly affected by heavy metal pretreatment (*P* ≤ 0.05). GST activity, measured as nmol glutathione conjugated min⁻¹ mg⁻¹ protein (± SEM), was highest in the control group (463 ± 11.50) and decreased following exposure to heavy metals, with nickel pretreatment reducing activity to 310 ± 3.60 and lead pretreatment further reducing it to 84 ± 4.50. Cytochrome P450 activity, expressed in equivalent units mg⁻¹ protein (± SEM), was 37 ± 2.87 in controls, slightly decreased to 29 ± 2.38 after nickel exposure, and increased to 52 ± 5.13 after lead pretreatment, with all differences being statistically significant. EST activity, expressed as nmol 1-naphthol min⁻¹ mg⁻¹ protein (± SEM), was 551 ± 44.42 in the control group, and decreased to 453 ± 11.93 and 324 ± 13.37 in nickel- and lead-pretreated mites, respectively. Different letters indicate statistically significant differences among treatments according to Tukey’s HSD test (*P* ≤ 0.05), and all values represent mean ± SEM of four biological replicates (*n* = 4). Percent reductions and mechanistic interpretations have been moved to the Discussion section (Table 2).

**Table 2:** Detoxification enzyme activities with pretreatment of selected heavy metals.

Treat	GST^b^ (CDNB conjugation)	Reduced activity (%)	P450s^a^ (TMBZ)	Reduced activity (%)	ESTs^c^ (α-Naphthyl Acetate)	Reduced activity (%)
Control	463^a^ (± 11.50)		37^a^ (± 2.87)		551^a^ (± 44.42)	
Nickel	310^b^ (± 3.60)	33%	29^ba^ (± 2.38)	22%	453^b^ (± 11.93)	18%
Lead	84^c^ (± 4.50)	82%	52^c^ (± 5.13)	**+** 40%	324^c^ (± 13.37)	42%

^a^equivalent units of cytochrome P450 mg^− 1^ protein (± SEM).

^b^nmol glutathione conjugated min^− 1^ mg^− 1^ protein (± SEM).

^c^nmol 1-naphthol min^− 1^ mg^− 1^ protein (± SEM).

## Discussion

The present study demonstrated that heavy metals can significantly modify the toxicity of pesticides in *R. robini*. Exposure to Pb and Ni increased the toxicity of propargite and chlorpyrifos, whereas an antagonistic interaction was observed with abamectin. These findings confirm that heavy metals are not only environmental contaminants but may also influence pesticide performance by altering physiological and biochemical detoxification processes in arthropods. Similar interactions between heavy metals and pesticides have been reported previously, where combined exposure resulted in greater toxicological effects than either stressor alone due to disruption of detoxification pathways and increased oxidative stress^21^. Heavy metals such as Pb and Cd can interfere with the activity of detoxification enzymes, including cytochrome P450 monooxygenases and glutathione S-transferases (GSTs), leading to altered xenobiotic metabolism and enhanced toxicity^22^.

The synergistic effects observed with propargite and chlorpyrifos are likely associated with impairment of detoxification mechanisms. Detoxification enzymes, particularly cytochrome P450s, GSTs, and esterases, play a central role in pesticide metabolism^23^. Previous studies have shown that heavy metals may inhibit or alter the activity of these enzymes, reducing the capacity of pests to detoxify pesticides and consequently increasing mortality^24^. Similarly, previous studies have demonstrated that inhibition of detoxification enzymes using synergists such as DEM, PBO, and TPP can significantly increase the sensitivity of *R. robini* to propargite, highlighting the central role of GSTs, cytochrome P450s, and esterases in modulating pesticide toxicity^25^. In the present study, GST activity was markedly reduced in mites exposed to Pb, suggesting a diminished ability to conjugate and eliminate toxic compounds. Similar reductions in GST activity have been reported in arthropods exposed to heavy metals, including the carabid beetle *Poecilus cupreus*, where dietary exposure to Zn and Cd significantly suppressed GST activity^26^. Because GSTs contribute to the detoxification of numerous xenobiotics, their inhibition may have increased the susceptibility of *R. robini* to propargite and chlorpyrifos.

Cytochrome P450 activity showed a different pattern, increasing significantly following Pb exposure. Although P450 enzymes are generally associated with detoxification, their induction under heavy metal stress may reflect a broader physiological response to environmental contaminants^27^. Previous studies have reported that heavy metals alter P450-mediated metabolic pathways and may redirect metabolic resources toward coping with metal stress. Transcriptomic analysis of the acarid mite *Aleurolyphus ovatus* demonstrated that Pb exposure significantly affected genes involved in cytochrome P450 metabolism, glutathione metabolism, and xenobiotic detoxification pathways^17^. In that study, high Pb concentrations stimulated the expression of detoxification-related genes, including GST and antioxidant enzymes, indicating substantial reorganization of metabolic pathways under metal stress. Such physiological adjustments may contribute to the altered pesticide susceptibility observed in the present study.

The antagonistic interaction observed between heavy metals and abamectin indicates that the effects of heavy metals are pesticide-specific. Similar antagonistic responses have been reported previously. Mehler et al. found that Pb exposure reduced the toxicity of the pyrethroid cypermethrin in *Chironomus dilutus* and suggested that changes in organismal sensitivity rather than pesticide bioavailability were responsible for the reduced toxicity^28^. Likewise, the reduced efficacy of abamectin in the present study may be associated with heavy metal-induced physiological adjustments that modify pesticide sensitivity. Interestingly, GST-mediated detoxification does not appear to be directly involved in abamectin metabolism in some arthropods. For example, studies on *Tetranychus urticae* indicated that GST enzymes are inhibited by abamectin and may function primarily in maintaining cellular homeostasis rather than directly detoxifying the acaricide^29^. This may explain why the changes in GST and P450 activity observed here do not fully account for the antagonistic response to abamectin, suggesting the involvement of additional mechanisms.

The present findings are consistent with previous reports showing that heavy metal–pesticide interactions may produce either synergistic or antagonistic outcomes depending on the compounds involved. Synergistic interactions have been reported for mixtures of heavy metals and pesticides in several invertebrates, including earthworms and silkworm larvae, where combined exposure resulted in toxicity greater than the sum of individual effects^21^. Conversely, antagonistic interactions have also been documented, indicating that heavy metals may either enhance or reduce pesticide efficacy through alterations in detoxification pathways, oxidative stress responses, and physiological sensitivity.

From an applied perspective, these findings suggest that heavy metal contamination may significantly influence pesticide performance in agricultural systems. The increased susceptibility of *R. robini* to propargite and chlorpyrifos in the presence of Pb and Ni indicates that pesticide efficacy in contaminated soils may differ from that in uncontaminated environments. In contrast, the reduced efficacy of abamectin suggests that heavy metal contamination could compromise the effectiveness of certain control measures. Therefore, environmental contamination should be considered when evaluating pesticide performance and developing pest management programs.

Although this study provides evidence that heavy metals alter pesticide susceptibility through changes in detoxification enzyme activity, several limitations should be acknowledged. The experiments were conducted under controlled laboratory conditions using a single concentration of each metal, and the molecular mechanisms underlying enzyme modulation remain unclear. Future studies should investigate environmentally relevant metal concentrations, chronic exposure effects, and the transcriptional regulation of detoxification pathways. Nevertheless, the present study demonstrates that heavy metals can substantially alter pesticide toxicity in *R. robini* and highlights the importance of considering environmental contaminants when assessing pesticide efficacy and resistance-related mechanisms.

Future research should focus on evaluating heavy metal–pesticide interactions under field conditions and across a broader range of environmentally relevant metal concentrations. Investigating the molecular basis of these interactions through transcriptomic, proteomic, and enzyme-kinetic approaches would improve our understanding of the mechanisms underlying altered pesticide susceptibility. In addition, long-term studies examining the effects of chronic exposure on reproduction, population growth, and resistance development in *R. robini* are needed to assess the ecological and pest management implications of heavy metal contamination in saffron production systems.

## Supplementary Information

Below is the link to the electronic supplementary material.

Supplementary Material 1

Supplementary Material 2

## Data Availability

All data generated or analysed during this study are included in this published article and its supplementary information files.
