# Supplementary material for: Combined toxicity of pesticides and heavy metals on Rhizoglyphus robini (Acari: Acaridae)
Source: Sci Rep. 2026 Jun 22;16:28575. doi: 10.1038/s41598-026-59265-5 (PMC13572370; doi:10.1038/s41598-026-59265-5)
Supplement: Supplementary file 1 [file 41598_2026_59265_MOESM1_ESM.pdf]

=Rhizoglyphus robini

PoloPlus Version 2.0
Date: 05 OCT 2024
Rhizoglyphus robini
Data file: C:\Users\Samadieh\Desktop\Cholor+Nickle.txt
Number of preparations: 2
Number of dose groups: 32
Model: Probit
Natural Response Parameter: no
Convert doses to logarithms: yes
LDs: 50
~~~~~~

Rhizoglyphus robini

Intercepts and slopes unconstrained. Preparation is (1) Color
Not estimating natural response

	parameter	standard error	t ratio
Color	0.336	0.067	4.983
SLOPE	4.165	0.348	11.974

Variance-Covariance matrix

	Color	SLOPE
Color	0.454292E-02	0.543560E-02
SLOPE	0.543560E-02	0.120983

Chi-squared goodness of fit test

prep	dose	n	r	expected	residual	probab	std resid
Color	1.800	27.	26.	24.82	1.184	0.919	0.836
	1.800	24.	22.	22.06	-0.059	0.919	-0.044
	1.800	38.	36.	34.93	1.074	0.919	0.639
	1.800	34.	32.	31.25	0.750	0.919	0.472
	1.200	35.	23.	26.15	-3.152	0.747	-1.226
	1.200	28.	21.	20.92	0.079	0.747	0.034
	1.200	26.	19.	19.43	-0.427	0.747	-0.193
	1.200	31.	23.	23.16	-0.163	0.747	-0.067
	0.800	22.	9.	10.41	-1.406	0.473	-0.600
	0.800	29.	14.	13.72	0.283	0.473	0.105
	0.800	26.	13.	12.30	0.702	0.473	0.276
	0.800	37.	15.	17.50	-2.501	0.473	-0.823
	0.500	39.	7.	6.99	0.006	0.179	0.003
	0.500	26.	7.	4.66	2.337	0.179	1.195
	0.500	20.	5.	3.59	1.413	0.179	0.824
	0.500	39.	6.	6.99	-0.994	0.179	-0.415

chi-square: 6.281   degrees of freedom: 14   heterogeneity: 0.449

Effective Doses

	dose	limits	0.90	0.95	0.99
LD50 Color	0.831	lower	0.780	0.770	0.750
		upper	0.882	0.892	0.912

Intercepts and slopes unconstrained. Preparation is (2) Col.Nick
Not estimating natural response

parameter	standard error	t ratio
~~~~~~

Col.Nick	0.582	0.078	7.463
SLOPE	3.830	0.369	10.385

Variance-Covariance matrix

	Col.Nick	SLOPE
Col.Nick	0.607585E-02	0.143445E-01
SLOPE	0.143445E-01	0.136010

Chi-squared goodness of fit test

prep	dose	n	r	expected	residual	probab	std resid
Col.Nick	1.500	23.	20.	20.60	-0.596	0.895	-0.406
	1.500	28.	26.	25.07	0.927	0.895	0.573
	1.500	33.	29.	29.55	-0.550	0.895	-0.313
	1.500	27.	25.	24.18	0.822	0.895	0.517
	0.000	31.	24.	22.31	1.691	0.720	0.676
	0.000	24.	18.	17.27	0.729	0.720	0.331
	0.000	24.	17.	17.27	-0.271	0.720	-0.123
	0.000	35.	22.	25.19	-3.187	0.720	-1.199
	0.660	27.	12.	12.32	-0.324	0.456	-0.125
	0.660	24.	10.	10.96	-0.955	0.456	-0.391
	0.660	29.	14.	13.24	0.763	0.456	0.284
	0.660	23.	11.	10.50	0.501	0.456	0.210
	0.440	21.	6.	4.55	1.452	0.217	0.769
	0.440	25.	4.	5.41	-1.415	0.217	-0.687
	0.440	28.	7.	6.06	0.935	0.217	0.429
	0.440	26.	5.	5.63	-0.631	0.217	-0.301

chi-square: 4.511 degrees of freedom: 14 heterogeneity: 0.322

Effective Doses

		dose	limits	0.90	0.95	0.99
LD50	Col.Nick	0.705	lower	0.655	0.646	0.626
			upper	0.754	0.763	0.783

HYPOTHESIS OF EQUALITY (equal slopes, equal intercepts): REJECTED (P<0.05)
(chi-square: 8.61, degrees of freedom: 2, tail probability: 0.013)

HYPOTHESIS OF PARALLELISM (equal slopes): NOT REJECTED (P>0.05)
(chi-square: 0.44, degrees of freedom: 1, tail probability: 0.508)

Lethal dose ratio (LD50)

	ratio	limits	0.95
Col.Nick	1.178	lower	1.054
		upper	1.317

=Rhizoglyphus robini

PoloPlus Version 2.0
Date: 05 OCT 2024
Rhizoglyphus robini
Data file: C:\Users\Samadieh\Desktop\Cholor+Nickle.txt
Number of preparations: 2
Number of dose groups: 32
Model: Probit
Natural Response Parameter: no
Convert doses to logarithms: yes
LDs: 50
~~~~~~

Rhizoglyphus robini
  Color   subjects 481   controls 93
    slope=4.165+-0.348   nat.resp.=0.000+-0.000
    heterogeneity=0.45
    LD50=0.831   95% limits: 0.770 to 0.892
  Col.Nick   subjects 428   controls 106
    slope=3.830+-0.369   nat.resp.=0.000+-0.000
    heterogeneity=0.32
    LD50=0.705   95% limits: 0.646 to 0.763
  HYPOTHESIS OF EQUALITY: Rejected
  HYPOTHESIS OF PARALLELISM: Not Rejected
  LETHAL DOSE RATIOS
    LD50
      Col.Nick ratio=1.178   95% limits: 1.054 to 1.317
~~~~~~

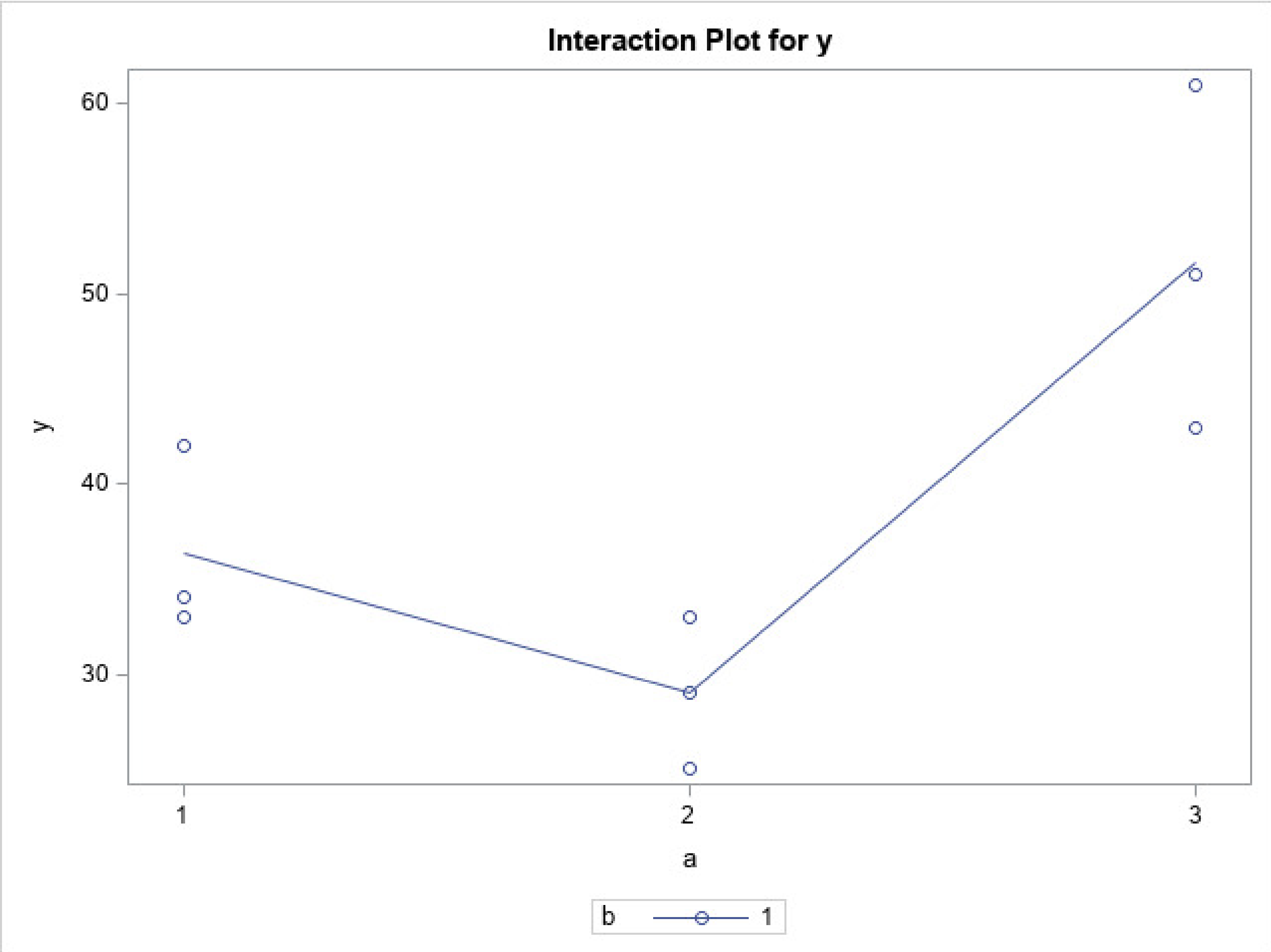
Interaction Plot for y
60
50
40
30
y
1
2
3
a
b
1

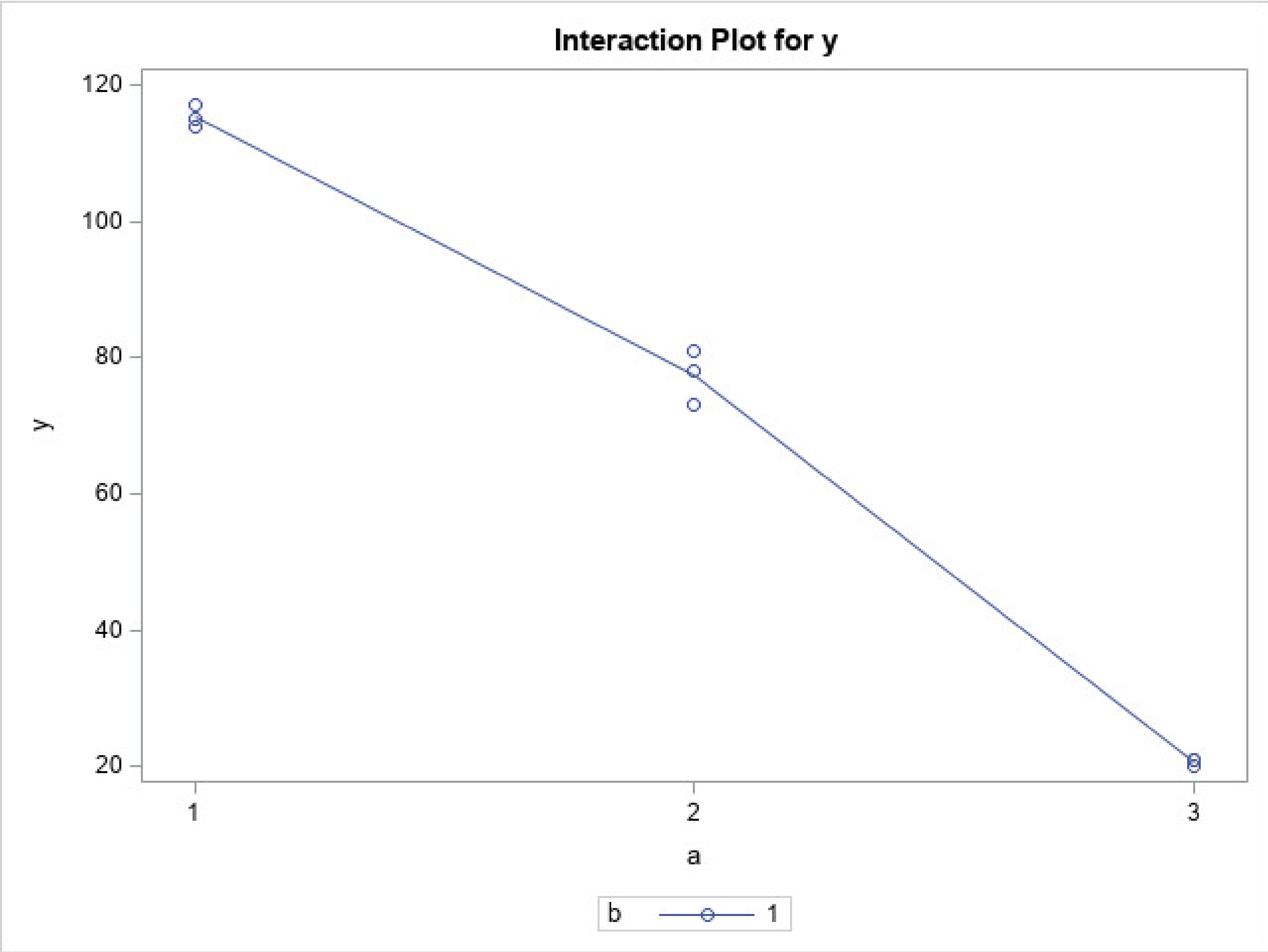
Interaction Plot for y
120
100
80
60
40
20
y
1
2
3
a
b
1

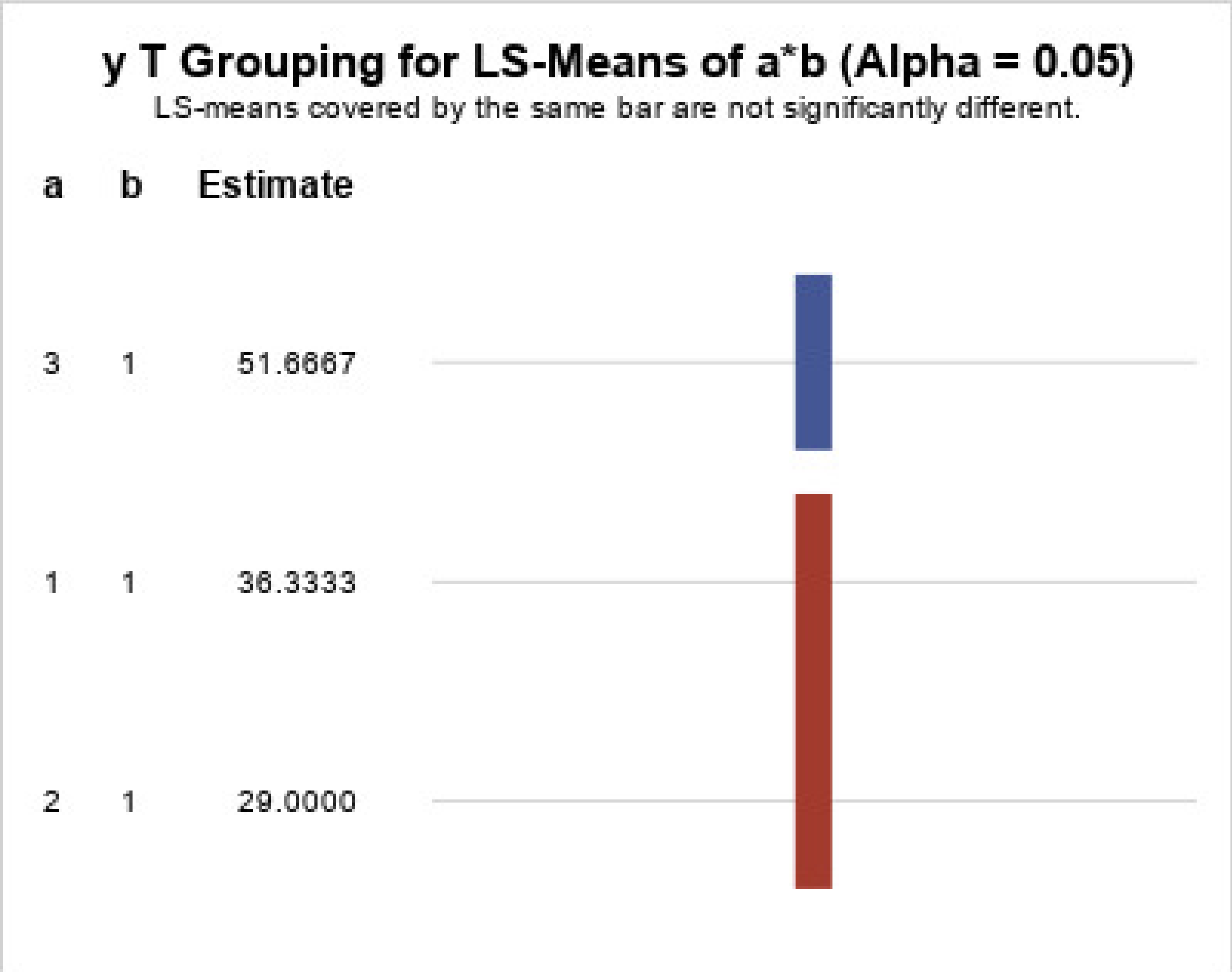
y T Grouping for LS-Means of a*b (Alpha = 0.05)
LS-means covered by the same bar are not significantly different.
a
b
Estimate
3
1
51.6667
1
1
36.3333
2
1
29.0000

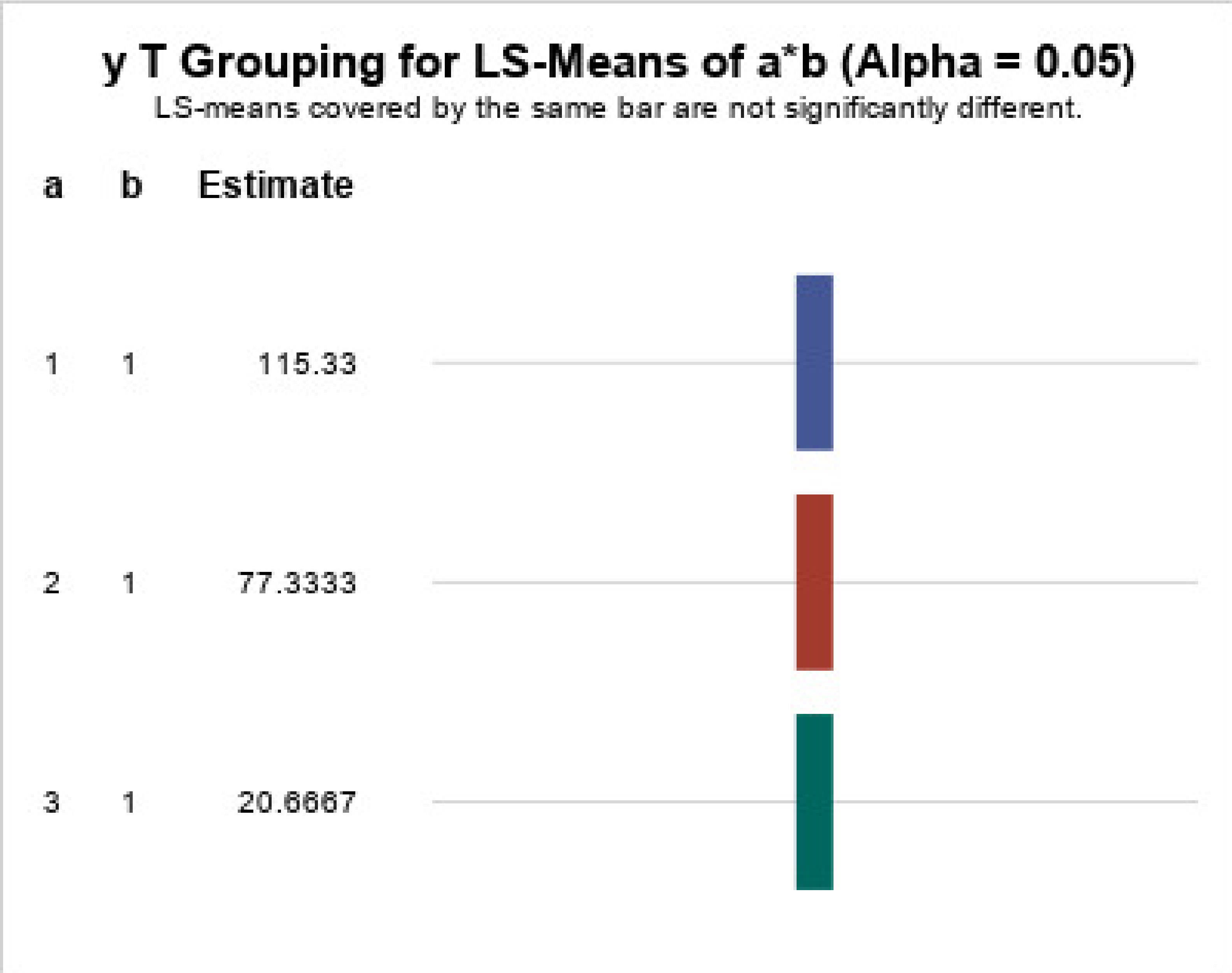
y T Grouping for LS-Means of a*b (Alpha = 0.05)
LS-means covered by the same bar are not significantly different.
a
b
Estimate
1
1
115.33
2
1
77.3333
3
1
20.6667

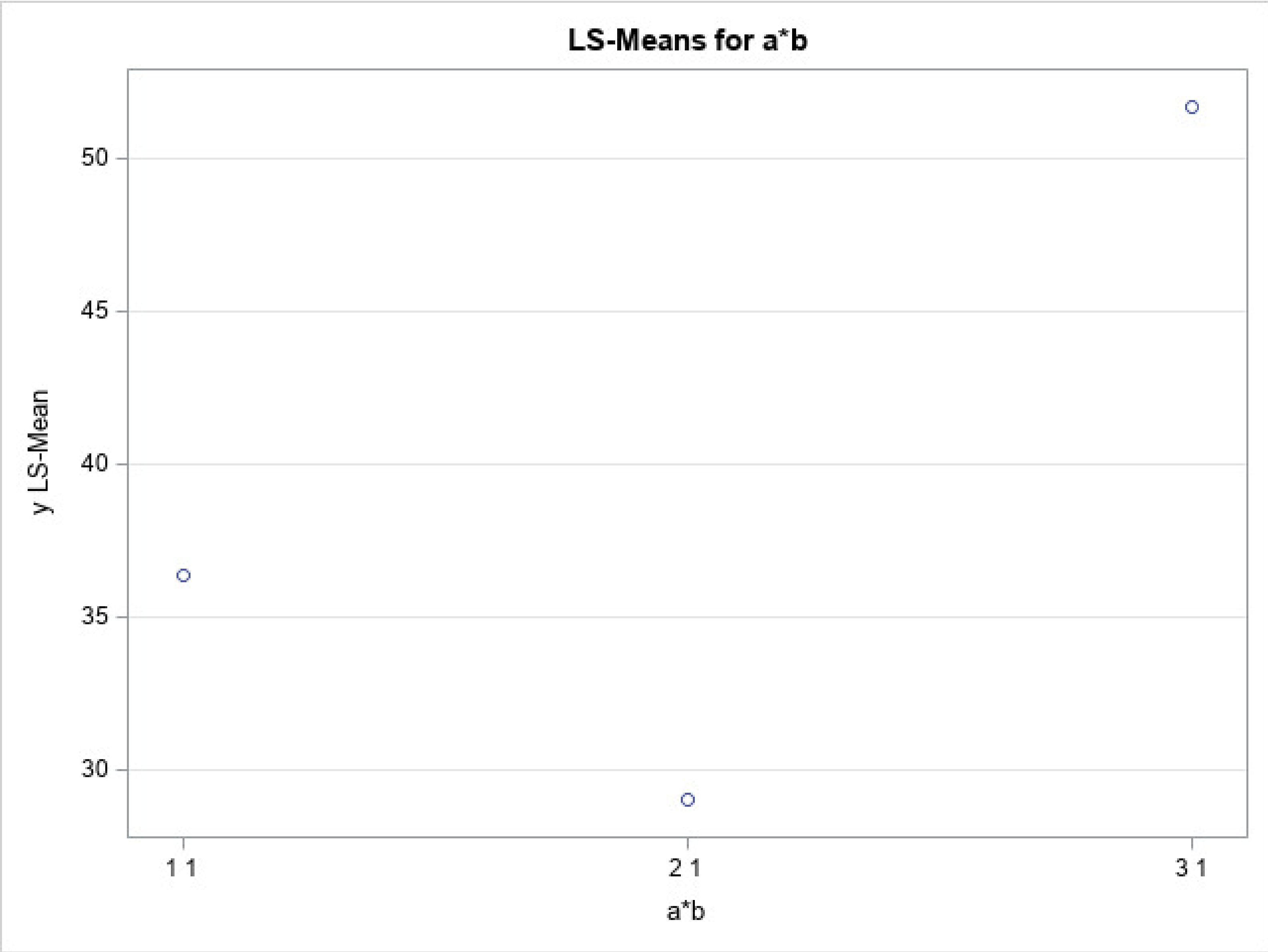
LS-Means for a*b
y LS-Mean
50
45
40
35
30
1 1
2 1
3 1
a*b

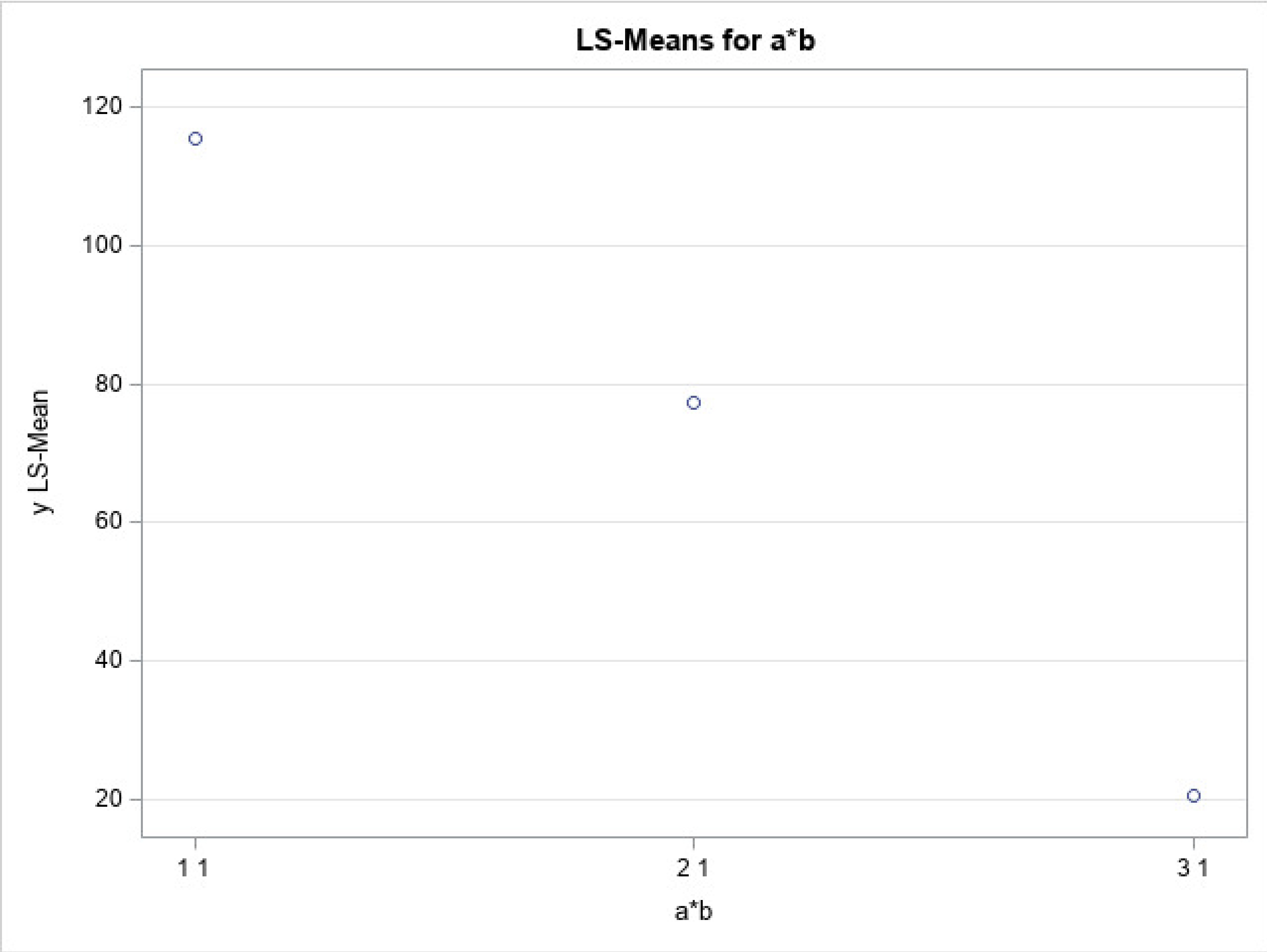
LS-Means for a*b
y LS-Mean
120
100
80
60
40
20
1 1
2 1
3 1
a*b

=Rhizoglyphus robini
*Propar
100 28 23
100 30 26
100 32 27
100 21 18
50 22 15
50 26 18
50 19 13
50 29 20
25 24 10
25 31 13
25 19 8
25 35 15
12.5 32 7
12.5 25 5
12.5 38 8
12.5 30 6
0 25 0
0 24 0
0 36 0
0 38 0
*Pro.lead
40 26 25
40 28 25
40 30 28
40 27 25
20 23 15
20 25 18
20 20 13
20 28 20
10 25 10
10 30 13
10 20 8
10 34 15
5 33 7
5 26 5
5 37 8
5 30 6
0 23 0
0 28 0
0 26 0
0 18 0

=Rhizoglyphus robini
*Propar
100 28 23
100 30 26
100 32 27
100 21 18
50 22 15
50 26 18
50 19 13
50 29 20
25 24 10
25 31 13
25 19 8
25 35 15
12.5 32 7
12.5 25 5
12.5 38 8
12.5 30 6
0 25 0
0 24 0
0 36 0
0 38 0
*Pro.Nick
60 26 23
60 29 26
60 30 27
60 20 18
30 23 15
30 24 18
30 20 13
30 28 20
15 25 10
15 30 13
15 20 8
15 34 15
7.5 35 7
7.5 28 5
7.5 34 8
7.5 32 6
0 25 0
0 27 0
0 22 0
0 26 0

=Rhizoglyphus robini

PoloPlus Version 2.0
Date: 05 OCT 2024
Rhizoglyphus robini
Data file: C:\Users\Samadieh\Desktop\Pro+lead.txt
Number of preparations: 2
Number of dose groups: 32
Model: Probit
Natural Response Parameter: no
Convert doses to logarithms: yes
LDs: 50
~~~~~~

Rhizoglyphus robini

Intercepts and slopes unconstrained. Preparation is (1) Pro
Not estimating natural response

	parameter	standard error	t ratio
Pro	-3.072	0.310	-9.900
SLOPE	2.065	0.201	10.286

Variance-Covariance matrix

	Pro	SLOPE
Pro	0.962652E-01	-0.608934E-01
SLOPE	-0.608934E-01	0.403035E-01

Chi-squared goodness of fit test

prep	dose	n	r	expected	residual	probab	std resid
Pro	100.000	28.	23.	23.94	-0.942	0.855	-0.506
	100.000	30.	26.	25.65	0.348	0.855	0.180
	100.000	32.	27.	27.36	-0.362	0.855	-0.182
	100.000	21.	18.	17.96	0.043	0.855	0.027
	50.000	22.	15.	14.72	0.285	0.669	0.129
	50.000	26.	18.	17.39	0.609	0.669	0.254
	50.000	19.	13.	12.71	0.291	0.669	0.142
	50.000	29.	20.	19.40	0.603	0.669	0.238
	25.000	24.	10.	10.24	-0.240	0.427	-0.099
	25.000	31.	13.	13.23	-0.227	0.427	-0.083
	25.000	19.	8.	8.11	-0.107	0.427	-0.050
	25.000	35.	15.	14.93	0.066	0.427	0.023
	12.500	32.	7.	6.72	0.280	0.210	0.122
	12.500	25.	5.	5.25	-0.250	0.210	-0.123
	12.500	38.	8.	7.98	0.020	0.210	0.008
	12.500	30.	6.	6.30	-0.300	0.210	-0.134

chi-square: 0.548 degrees of freedom: 14 heterogeneity: 0.039

Effective Doses

	dose	limits	0.90	0.95	0.99
LD50 Pro	30.721	lower	27.164	26.501	25.207
		upper	34.646	35.469	37.173

Intercepts and slopes unconstrained. Preparation is (2) Pro.Lead
Not estimating natural response

parameter standard error t ratio
~~~~~~

Pro.Lead	-2.589	0.244	-10.612
SLOPE	2.440	0.215	11.324

Variance-Covariance matrix

	Pro.Lead	SLOPE
Pro.Lead	0.595173E-01	-0.505163E-01
SLOPE	-0.505163E-01	0.464298E-01

Chi-squared goodness of fit test

prep	dose	n	r	expected	residual	probab	std resid
Pro.Lead	40.000	26.	25.	23.57	1.428	0.907	0.963
	40.000	28.	25.	25.38	-0.385	0.907	-0.250
	40.000	30.	28.	27.20	0.802	0.907	0.503
	40.000	27.	25.	24.48	0.522	0.907	0.345
	20.000	23.	15.	16.58	-1.581	0.721	-0.735
	20.000	25.	18.	18.02	-0.023	0.721	-0.010
	20.000	20.	13.	14.42	-1.419	0.721	-0.707
	20.000	28.	20.	20.19	-0.186	0.721	-0.078
	10.000	25.	10.	11.02	-1.020	0.441	-0.411
	10.000	30.	13.	13.22	-0.224	0.441	-0.082
	10.000	20.	8.	8.82	-0.816	0.441	-0.368
	10.000	34.	15.	14.99	0.013	0.441	0.004
	5.000	33.	7.	6.22	0.780	0.188	0.347
	5.000	26.	5.	4.90	0.099	0.188	0.050
	5.000	37.	8.	6.97	1.026	0.188	0.431
	5.000	30.	6.	5.65	0.345	0.188	0.161

chi-square: 3.054 degrees of freedom: 14 heterogeneity: 0.218

Effective Doses

		dose	limits	0.90	0.95	0.99
LD50	Pro.Lead	11.509	lower	10.335	10.115	9.685
			upper	12.782	13.045	13.584

HYPOTHESIS OF EQUALITY (equal slopes, equal intercepts): REJECTED (P<0.05)
(chi-square: 81.67, degrees of freedom: 2, tail probability: 0.000)

HYPOTHESIS OF PARALLELISM (equal slopes): NOT REJECTED (P>0.05)
(chi-square: 1.63, degrees of freedom: 1, tail probability: 0.202)

Lethal dose ratio (LD50)

	ratio	limits	0.95
Pro.Lead	2.669	lower	2.198
		upper	3.241

```
=Rhizoglyphus robini

PoloPlus  Version 2.0
Date: 05 OCT 2024
Rhizoglyphus robini
Data file: C:\Users\Samadieh\Desktop\Pro+lead.txt
Number of preparations: 2
Number of dose groups: 32
Model: Probit
Natural Response Parameter: no
Convert doses to logarithms: yes
LDs:  50
~~~~~~~~~~~~~~~~~~~~~~~~~~~~~~~~~~~~~~~~~~~~~~~~~~~~~~~~~~~~~~~~~~~~~~~~~~~~~~~~~~~~~

Rhizoglyphus robini
  Pro        subjects 441   controls 123
    slope=2.065+-0.201   nat.resp.=0.000+-0.000
    heterogeneity=0.04
    LD50=30.721    95% limits: 26.501 to 35.469
  Pro.Lead   subjects 442   controls 95
    slope=2.440+-0.215   nat.resp.=0.000+-0.000
    heterogeneity=0.22
    LD50=11.509    95% limits: 10.115 to 13.045
  HYPOTHESIS OF EQUALITY:  Rejected
  HYPOTHESIS OF PARALLELISM:  Not Rejected
  LETHAL DOSE RATIOS
    LD50
      Pro.Lead  ratio=2.669    95% limits: 2.198 to 3.241
```

=Rhizoglyphus robini

PoloPlus Version 2.0
Date: 30 APR 2026
Rhizoglyphus robini
Data file: C:\Users\Hosein\Desktop\Pro+Nickle.txt
Number of preparations: 2
Number of dose groups: 32
Model: Probit
Natural Response Parameter: no
Convert doses to logarithms: yes
LDs: 50
~~~~~~

Rhizoglyphus robini

Intercepts and slopes unconstrained. Preparation is (1) Propar
Not estimating natural response

	parameter	standard error	t ratio
Propar	-3.072	0.310	-9.900
SLOPE	2.065	0.201	10.286

Variance-Covariance matrix

	Propar	SLOPE
Propar	0.962652E-01	-0.608934E-01
SLOPE	-0.608934E-01	0.403035E-01

Chi-squared goodness of fit test

prep	dose	n	r	expected	residual	probab	std resid
Propar	100.000	28.	23.	23.94	-0.942	0.855	-0.506
	100.000	30.	26.	25.65	0.348	0.855	0.180
	100.000	32.	27.	27.36	-0.362	0.855	-0.182
	100.000	21.	18.	17.96	0.043	0.855	0.027
	50.000	22.	15.	14.72	0.285	0.669	0.129
	50.000	26.	18.	17.39	0.609	0.669	0.254
	50.000	19.	13.	12.71	0.291	0.669	0.142
	50.000	29.	20.	19.40	0.603	0.669	0.238
	25.000	24.	10.	10.24	-0.240	0.427	-0.099
	25.000	31.	13.	13.23	-0.227	0.427	-0.083
	25.000	19.	8.	8.11	-0.107	0.427	-0.050
	25.000	35.	15.	14.93	0.066	0.427	0.023
	12.500	32.	7.	6.72	0.280	0.210	0.122
	12.500	25.	5.	5.25	-0.250	0.210	-0.123
	12.500	38.	8.	7.98	0.020	0.210	0.008
	12.500	30.	6.	6.30	-0.300	0.210	-0.134

chi-square: 0.548   degrees of freedom: 14   heterogeneity: 0.039

Effective Doses

		dose	limits	0.90	0.95	0.99
LD50	Propar	30.721	lower	27.164	26.501	25.207
			upper	34.646	35.469	37.173

Intercepts and slopes unconstrained. Preparation is (2) Pro.Nick
Not estimating natural response

parameter   standard error   t ratio
~~~~~~

Pro.Nick	-2.880	0.275	-10.458
SLOPE	2.306	0.211	10.937

Variance-Covariance matrix

	Pro.Nick	SLOPE
Pro.Nick	0.758360E-01	-0.563276E-01
SLOPE	-0.563276E-01	0.444560E-01

Chi-squared goodness of fit test

prep	dose	n	r	expected	residual	probab	std resid
Pro.Nick	60.000	26.	23.	23.11	-0.110	0.889	-0.069
	60.000	29.	26.	25.78	0.223	0.889	0.132
	60.000	30.	27.	26.67	0.334	0.889	0.194
	60.000	20.	18.	17.78	0.223	0.889	0.158
	30.000	23.	15.	16.12	-1.116	0.701	-0.508
	30.000	24.	18.	16.82	1.184	0.701	0.528
	30.000	20.	13.	14.01	-1.014	0.701	-0.495
	30.000	28.	20.	19.62	0.381	0.701	0.157
	15.000	25.	10.	10.83	-0.834	0.433	-0.337
	15.000	30.	13.	13.00	-0.001	0.433	0.000
	15.000	20.	8.	8.67	-0.667	0.433	-0.301
	15.000	34.	15.	14.73	0.266	0.433	0.092
	7.500	35.	7.	6.80	0.198	0.194	0.084
	7.500	28.	5.	5.44	-0.442	0.194	-0.211
	7.500	34.	8.	6.61	1.392	0.194	0.603
	7.500	32.	6.	6.22	-0.219	0.194	-0.098

chi-square: 1.529 degrees of freedom: 14 heterogeneity: 0.109

Effective Doses

		dose	limits	0.90	0.95	0.99
LD50	Pro.Nick	17.736	lower	15.854	15.503	14.820
			upper	19.804	20.235	21.123

HYPOTHESIS OF EQUALITY (equal slopes, equal intercepts): REJECTED (P<0.05)
(chi-square: 29.24, degrees of freedom: 2, tail probability: 0.000)

HYPOTHESIS OF PARALLELISM (equal slopes): NOT REJECTED (P>0.05)
(chi-square: 0.69, degrees of freedom: 1, tail probability: 0.408)

Lethal dose ratio (LD50)

	ratio	limits	0.95
Pro.Nick	1.732	lower	1.421
		upper	2.111

=Rhizoglyphus robini

PoloPlus Version 2.0
Date: 30 APR 2026
Rhizoglyphus robini
Data file: C:\Users\Hosein\Desktop\Pro+Nickle.txt
Number of preparations: 2
Number of dose groups: 32
Model: Probit
Natural Response Parameter: no
Convert doses to logarithms: yes
LDs: 50
~~~~~~

Rhizoglyphus robini
  Propar   subjects 441   controls 123
    slope=2.065+-0.201   nat.resp.=0.000+-0.000
    heterogeneity=0.04
    LD50=30.721   95% limits: 26.501 to 35.469
  Pro.Nick   subjects 438   controls 100
    slope=2.306+-0.211   nat.resp.=0.000+-0.000
    heterogeneity=0.11
    LD50=17.736   95% limits: 15.503 to 20.235
  HYPOTHESIS OF EQUALITY: Rejected
  HYPOTHESIS OF PARALLELISM: Not Rejected
  LETHAL DOSE RATIOS
    LD50
      Pro.Nick ratio=1.732   95% limits: 1.421 to 2.111
~~~~~~

The SAS System

The GLM Procedure

Class Level Information		
**Class**	**Levels**	**Values**
**a**	3	1 2 3
**b**	1	1

**Number of Observations Read**	9
**Number of Observations Used**	9

The SAS System

The GLM Procedure

Dependent Variable: y

Source	DF	Sum of Squares	Mean Square	F Value	Pr > F
Model	2	77753.55556	38876.77778	17.07	0.0033
Error	6	13668.66667	2278.11111		
Corrected Total	8	91422.22222			

R-Square	Coeff Var	Root MSE	y Mean
0.850489	10.78499	47.72956	442.5556

Source	DF	Type III SS	Mean Square	F Value	Pr > F
a	2	77753.55556	38876.77778	17.07	0.0033
b	0	0.00000	.	.	.
a*b	0	0.00000	.	.	.

Interaction Plot for y by a

The SAS System

The GLM Procedure

Distribution of y by a*b

Level of a	Level of b	N	y	
			Mean	Std Dev
1	1	3	551.000000	76.6224510
2	1	3	452.666667	20.5020324
3	1	3	324.000000	23.3023604

The SAS System

The GLM Procedure
Least Squares Means

a	b	y LSMEAN
1	1	551.000000
2	1	452.666667
3	1	324.000000

Plot of y least-squares means for a*b.

LinesPlot.png

The SAS System

The GLM Procedure

Class Level Information		
**Class**	**Levels**	**Values**
**a**	3	1 2 3
**b**	1	1

**Number of Observations Read**	9
**Number of Observations Used**	9

The SAS System

The GLM Procedure

Dependent Variable: y

Source	DF	Sum of Squares	Mean Square	F Value	Pr > F
Model	2	802.666667	401.333333	9.90	0.0126
Error	6	243.333333	40.555556		
Corrected Total	8	1046.000000			

R-Square	Coeff Var	Root MSE	y Mean
0.767368	16.32904	6.368324	39.00000

Source	DF	Type III SS	Mean Square	F Value	Pr > F
a	2	802.6666667	401.3333333	9.90	0.0126
b	0	0.0000000	.	.	.
a*b	0	0.0000000	.	.	.

Interaction Plot for y by a

The SAS System

The GLM Procedure

Distribution of y by a*b

Level of a	Level of b	N	y Mean	y Std Dev
1	1	3	36.3333333	4.93288286
2	1	3	29.0000000	4.00000000
3	1	3	51.6666667	9.01849951

The SAS System

The GLM Procedure
Least Squares Means

a	b	y LSMEAN
1	1	36.3333333
2	1	29.0000000
3	1	51.6666667

Plot of y least-squares means for a*b.

LinesPlot.png

The SAS System

The GLM Procedure

Class Level Information		
**Class**	**Levels**	**Values**
a	3	1 2 3
b	1	1

Number of Observations Read	9
Number of Observations Used	9

The SAS System

The GLM Procedure

Dependent Variable: y

Source	DF	Sum of Squares	Mean Square	F Value	Pr > F
Model	2	13616.88889	6808.44444	1075.02	<.0001
Error	6	38.00000	6.33333		
Corrected Total	8	13654.88889			

R-Square	Coeff Var	Root MSE	y Mean
0.997217	3.538985	2.516611	71.11111

Source	DF	Type III SS	Mean Square	F Value	Pr > F
a	2	13616.88889	6808.44444	1075.02	<.0001
b	0	0.00000	.	.	.
a*b	0	0.00000	.	.	.

Interaction Plot for y by a

The SAS System

The GLM Procedure

Distribution of y by a*b

Level of a	Level of b	N	y	
			Mean	Std Dev
1	1	3	115.333333	1.52752523
2	1	3	77.333333	4.04145188
3	1	3	20.666667	0.57735027

The SAS System

The GLM Procedure
Least Squares Means

a	b	y LSMEAN
1	1	115.333333
2	1	77.333333
3	1	20.666667

Plot of y least-squares means for a*b.

LinesPlot1.png

The SAS System

The GLM Procedure

Class Level Information		
Class	Levels	Values
a	3	1 2 3
b	1	1

Number of Observations Read	9
Number of Observations Used	9

The SAS System

The GLM Procedure

Dependent Variable: y

Source	DF	Sum of Squares	Mean Square	F Value	Pr > F
Model	2	802.666667	401.333333	9.90	0.0126
Error	6	243.333333	40.555556		
Corrected Total	8	1046.000000			

R-Square	Coeff Var	Root MSE	y Mean
0.767368	16.32904	6.368324	39.00000

Source	DF	Type III SS	Mean Square	F Value	Pr > F
a	2	802.6666667	401.3333333	9.90	0.0126
b	0	0.0000000	.	.	.
a*b	0	0.0000000	.	.	.

Interaction Plot for y by a

The SAS System

The GLM Procedure

Distribution of y by a*b

Level of a	Level of b	N	y	
			Mean	Std Dev
1	1	3	36.3333333	4.93288286
2	1	3	29.0000000	4.00000000
3	1	3	51.6666667	9.01849951

The SAS System

The GLM Procedure
Least Squares Means

a	b	y LSMEAN
1	1	36.3333333
2	1	29.0000000
3	1	51.6666667

Plot of y least-squares means for a*b.

LinesPlot.png

=Rhizoglyphus robini
*Pro.ZAV
6.4 33 27
6.4 21 18
6.4 22 18
4.3 20 15
4.3 23 17
4.3 28 19
4.3 26 16
2.9 28 12
2.9 19 7
2.9 30 15
2.9 29 14
1.9 27 7
1.9 25 6
1.9 28 8
1.9 29 5
0 21 0
0 26 0
0 24 0
0 20 0
*Aba.DEM
5.4 32 28
5.4 30 25
5.4 25 22
5.4 28 24
3.6 26 18
3.6 34 24
3.6 42 28
3.6 31 22
2.4 35 17
2.4 35 16
2.4 30 15
2.4 29 14
1.6 27 6
1.6 25 5
1.6 28 6
1.6 29 7
0 23 0
0 21 0
0 30 0
0 21 0

=Rhizoglyphus robini

PoloPlus Version 2.0
Date: 30 APR 2026
Rhizoglyphus robini
Data file: C:\Users\Hosein\Desktop\Aba+lead.txt
Number of preparations: 2
Number of dose groups: 31
Model: Probit
Natural Response Parameter: no
Convert doses to logarithms: yes
LDs: 50
~~~~~~

Rhizoglyphus robini

Intercepts and slopes unconstrained. Preparation is (1) Aba
Not estimating natural response

	parameter	standard error	t ratio
Aba	-1.425	0.169	-8.425
SLOPE	3.467	0.345	10.044

Variance-Covariance matrix

	Aba	SLOPE
Aba	0.285887E-01	-0.543269E-01
SLOPE	-0.543269E-01	0.119121

Chi-squared goodness of fit test

prep	dose	n	r	expected	residual	probab	std resid
Aba	5.400	32.	28.	27.76	0.241	0.867	0.126
	5.400	30.	25.	26.02	-1.024	0.867	-0.551
	5.400	25.	22.	21.69	0.313	0.867	0.185
	5.400	28.	24.	24.29	-0.289	0.867	-0.161
	3.600	26.	18.	18.01	-0.015	0.693	-0.006
	3.600	34.	24.	23.56	0.442	0.693	0.164
	3.600	42.	28.	29.10	-1.101	0.693	-0.368
	3.600	31.	22.	21.48	0.521	0.693	0.203
	2.400	35.	17.	16.02	0.983	0.458	0.334
	2.400	35.	16.	16.02	-0.017	0.458	-0.006
	2.400	30.	15.	13.73	1.271	0.458	0.466
	2.400	29.	14.	13.27	0.729	0.458	0.272
	1.600	27.	6.	6.39	-0.391	0.237	-0.177
	1.600	25.	5.	5.92	-0.918	0.237	-0.432
	1.600	28.	6.	6.63	-0.628	0.237	-0.279
	1.600	29.	7.	6.86	0.135	0.237	0.059

chi-square: 1.285   degrees of freedom: 14   heterogeneity: 0.092

Effective Doses

	dose	limits	0.90	0.95	0.99
LD50 Aba	2.576	lower	2.393	2.357	2.284
		upper	2.756	2.792	2.863

Intercepts and slopes unconstrained. Preparation is (2) Aba+Lead
Not estimating natural response

parameter   standard error   t ratio
~~~~~~

Aba+Lead	-1.602	0.204	-7.843
SLOPE	3.231	0.376	8.591

Variance-Covariance matrix

	Aba+Lead	SLOPE
Aba+Lead	0.417462E-01	-0.724602E-01
SLOPE	-0.724602E-01	0.141479

Chi-squared goodness of fit test

prep	dose	n	r	expected	residual	probab	std resid
Aba+Lead	6.400	33.	27.	27.79	-0.785	0.842	-0.375
	6.400	21.	18.	17.68	0.318	0.842	0.191
	6.400	22.	18.	18.52	-0.523	0.842	-0.306
	4.300	20.	15.	13.43	1.567	0.672	0.746
	4.300	23.	17.	15.45	1.552	0.672	0.689
	4.300	28.	19.	18.81	0.193	0.672	0.078
	4.300	26.	16.	17.46	-1.464	0.672	-0.611
	2.900	28.	12.	12.79	-0.793	0.457	-0.301
	2.900	19.	7.	8.68	-1.681	0.457	-0.774
	2.900	30.	15.	13.71	1.293	0.457	0.474
	2.900	29.	14.	13.25	0.750	0.457	0.280
	1.900	27.	7.	6.52	0.481	0.241	0.216
	1.900	25.	6.	6.04	-0.036	0.241	-0.017
	1.900	28.	8.	6.76	1.239	0.241	0.547
	1.900	29.	5.	7.00	-2.002	0.241	-0.869

chi-square: 3.775 degrees of freedom: 13 heterogeneity: 0.290

Effective Doses

	dose	limits	0.90	0.95	0.99
LD50 Aba+Lead	3.133	lower	2.883	2.834	2.736
		upper	3.394	3.448	3.560

HYPOTHESIS OF EQUALITY (equal slopes, equal intercepts): REJECTED (P<0.05)
(chi-square: 10.16, degrees of freedom: 2, tail probability: 0.006)

HYPOTHESIS OF PARALLELISM (equal slopes): NOT REJECTED (P>0.05)
(chi-square: 0.21, degrees of freedom: 1, tail probability: 0.645)

Lethal dose ratio (LD50)

	ratio	limits	0.95
Aba+Lead	0.822	lower	0.723
		upper	0.936

=Rhizoglyphus robini

PoloPlus Version 2.0
Date: 30 APR 2026
Rhizoglyphus robini
Data file: C:\Users\Hosein\Desktop\Aba+lead.txt
Number of preparations: 2
Number of dose groups: 31
Model: Probit
Natural Response Parameter: no
Convert doses to logarithms: yes
LDs: 50
~~~~~~

Rhizoglyphus robini
  Aba   subjects 486   controls 95
    slope=3.467+-0.345   nat.resp.=0.000+-0.000
    heterogeneity=0.09
    LD50=2.576   95% limits: 2.357 to 2.792
  Aba+Lead   subjects 388   controls 91
    slope=3.231+-0.376   nat.resp.=0.000+-0.000
    heterogeneity=0.29
    LD50=3.133   95% limits: 2.834 to 3.448
  HYPOTHESIS OF EQUALITY: Rejected
  HYPOTHESIS OF PARALLELISM: Not Rejected
  LETHAL DOSE RATIOS
    LD50
      Aba+Lead ratio=0.822   95% limits: 0.723 to 0.936
~~~~~~

=Rhizoglyphus robini

PoloPlus Version 2.0
Date: 30 APR 2026
Rhizoglyphus robini
Data file: C:\Users\Hosein\Desktop\Aba+Nickle.txt
Number of preparations: 2
Number of dose groups: 32
Model: Probit
Natural Response Parameter: no
Convert doses to logarithms: yes
LDs: 50
~~~~~~

Rhizoglyphus robini

Intercepts and slopes unconstrained. Preparation is (1) Aba
Not estimating natural response

	parameter	standard error	t ratio
Aba	-1.425	0.169	-8.425
SLOPE	3.467	0.345	10.044

Variance-Covariance matrix

	Aba	SLOPE
Aba	0.285887E-01	-0.543269E-01
SLOPE	-0.543269E-01	0.119121

Chi-squared goodness of fit test

prep	dose	n	r	expected	residual	probab	std resid
Aba	5.400	32.	28.	27.76	0.241	0.867	0.126
	5.400	30.	25.	26.02	-1.024	0.867	-0.551
	5.400	25.	22.	21.69	0.313	0.867	0.185
	5.400	28.	24.	24.29	-0.289	0.867	-0.161
	3.600	26.	18.	18.01	-0.015	0.693	-0.006
	3.600	34.	24.	23.56	0.442	0.693	0.164
	3.600	42.	28.	29.10	-1.101	0.693	-0.368
	3.600	31.	22.	21.48	0.521	0.693	0.203
	2.400	35.	17.	16.02	0.983	0.458	0.334
	2.400	35.	16.	16.02	-0.017	0.458	-0.006
	2.400	30.	15.	13.73	1.271	0.458	0.466
	2.400	29.	14.	13.27	0.729	0.458	0.272
	1.600	27.	6.	6.39	-0.391	0.237	-0.177
	1.600	25.	5.	5.92	-0.918	0.237	-0.432
	1.600	28.	6.	6.63	-0.628	0.237	-0.279
	1.600	29.	7.	6.86	0.135	0.237	0.059

chi-square: 1.285 degrees of freedom: 14 heterogeneity: 0.092

Effective Doses

	dose	limits	0.90	0.95	0.99
LD50 Aba	2.576	lower	2.393	2.357	2.284
		upper	2.756	2.792	2.863

Intercepts and slopes unconstrained. Preparation is (2) Aba+Nick
Not estimating natural response

parameter standard error t ratio
~~~~~~

Aba+Nick	-1.428	0.194	-7.369
SLOPE	3.016	0.354	8.524

Variance-Covariance matrix

	Aba+Nick	SLOPE
Aba+Nick	0.375638E-01	-0.645347E-01
SLOPE	-0.645347E-01	0.125236

Chi-squared goodness of fit test

prep	dose	n	r	expected	residual	probab	std resid
Aba+Nick	6.000	30.	23.	24.63	-1.629	0.821	-0.776
	6.000	27.	21.	22.17	-1.166	0.821	-0.585
	6.000	28.	23.	22.99	0.013	0.821	0.006
	6.000	21.	16.	17.24	-1.240	0.821	-0.706
	4.000	23.	17.	14.97	2.028	0.651	0.887
	4.000	31.	22.	20.18	1.821	0.651	0.686
	4.000	28.	20.	18.23	1.773	0.651	0.703
	4.000	27.	19.	17.58	1.424	0.651	0.575
	2.700	24.	11.	10.79	0.213	0.449	0.087
	2.700	30.	13.	13.48	-0.484	0.449	-0.178
	2.700	25.	11.	11.24	-0.237	0.449	-0.095
	2.700	23.	10.	10.34	-0.338	0.449	-0.142
	1.800	22.	5.	5.61	-0.615	0.255	-0.301
	1.800	27.	7.	6.89	0.109	0.255	0.048
	1.800	25.	6.	6.38	-0.380	0.255	-0.175
	1.800	23.	5.	5.87	-0.870	0.255	-0.416

chi-square: 3.890 degrees of freedom: 14 heterogeneity: 0.278

Effective Doses

		dose	limits	0.90	0.95	0.99
LD50	Aba+Nick	2.975	lower	2.719	2.668	2.564
			upper	3.231	3.282	3.386

HYPOTHESIS OF EQUALITY (equal slopes, equal intercepts): REJECTED (P<0.05)
(chi-square: 6.82, degrees of freedom: 2, tail probability: 0.033)

HYPOTHESIS OF PARALLELISM (equal slopes): NOT REJECTED (P>0.05)
(chi-square: 0.83, degrees of freedom: 1, tail probability: 0.362)

Lethal dose ratio (LD50)

	ratio	limits	0.95
Aba+Nick	0.866	lower	0.758
		upper	0.989

=Rhizoglyphus robini

PoloPlus Version 2.0
Date: 30 APR 2026
Rhizoglyphus robini
Data file: C:\Users\Hosein\Desktop\Aba+Nickle.txt
Number of preparations: 2
Number of dose groups: 32
Model: Probit
Natural Response Parameter: no
Convert doses to logarithms: yes
LDs: 50
~~~~~~

Rhizoglyphus robini
  Aba   subjects 486   controls 95
    slope=3.467+-0.345   nat.resp.=0.000+-0.000
    heterogeneity=0.09
    LD50=2.576   95% limits: 2.357 to 2.792
  Aba+Nick   subjects 414   controls 101
    slope=3.016+-0.354   nat.resp.=0.000+-0.000
    heterogeneity=0.28
    LD50=2.975   95% limits: 2.668 to 3.282
  HYPOTHESIS OF EQUALITY: Rejected
  HYPOTHESIS OF PARALLELISM: Not Rejected
  LETHAL DOSE RATIOS
    LD50
      Aba+Nick ratio=0.866   95% limits: 0.758 to 0.989
~~~~~~

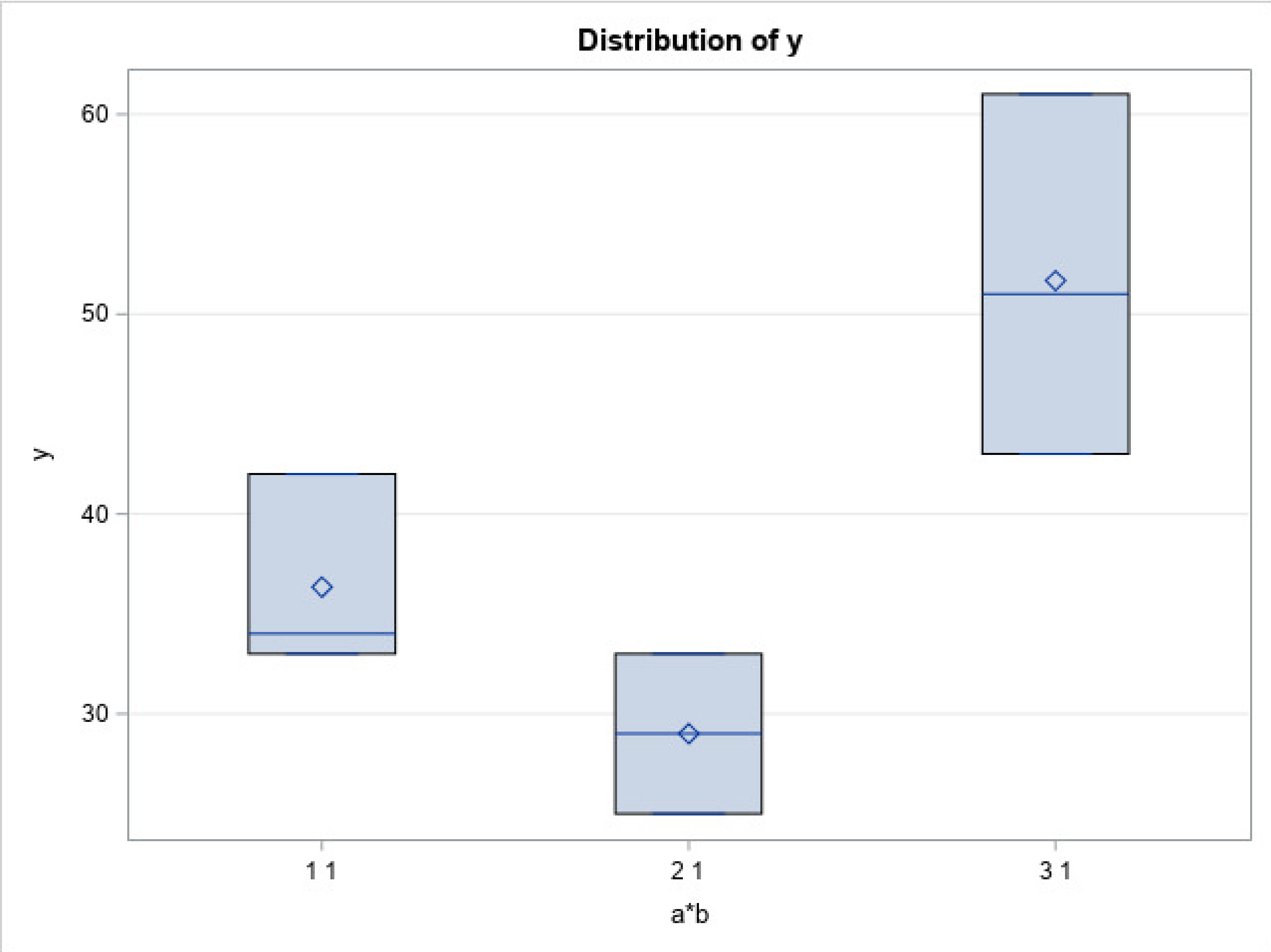
Distribution of y
60
50
40
30
y
1 1
2 1
3 1
a*b

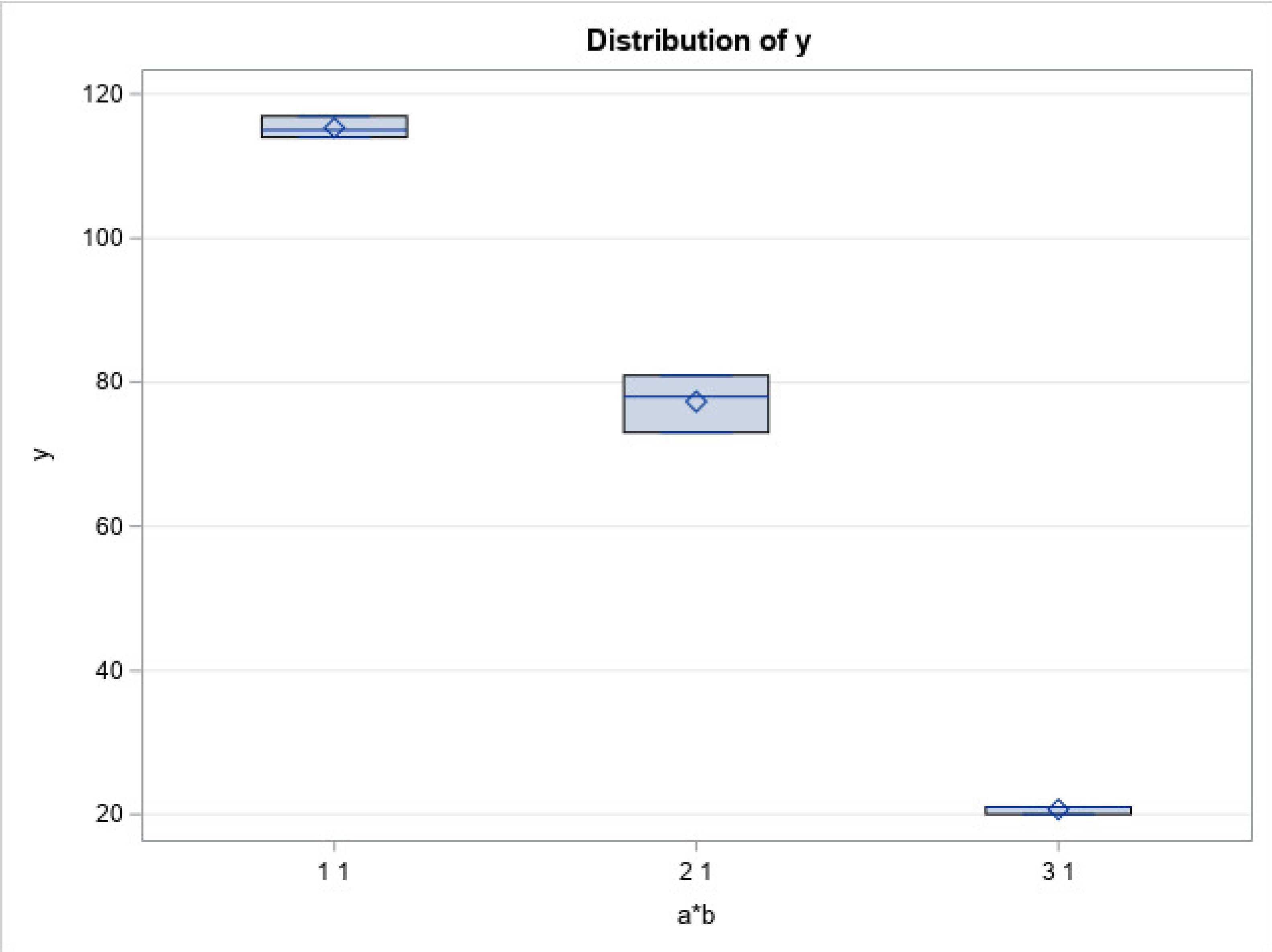
Distribution of y
120
100
80
60
40
20
y
1 1
2 1
3 1
a*b

=Rhizoglyphus robini
*Color
1.8 27 26
1.8 24 22
1.8 38 36
1.8 34 32
1.2 35 23
1.2 28 21
1.2 26 19
1.2 31 23
0.8 22 9
0.8 29 14
0.8 26 13
0.8 37 15
0.5 39 7
0.5 26 7
0.5 20 5
0.5 39 6
0 27 0
0 22 0
0 20 0
0 24 0
*Colo.lead
1.2 23 22
1.2 28 26
1.2 30 28
1.2 26 25
0.8 27 20
0.8 23 18
0.8 28 19
0.8 23 17
0.5 28 14
0.5 24 12
0.5 21 11
0.5 30 14
0.3 25 8
0.3 29 7
0.3 24 8
0.3 26 7
0 22 0
0 29 0
0 23 0
0 21 0

=Rhizoglyphus robini
*Color
1.8 27 26
1.8 24 22
1.8 38 36
1.8 34 32
1.2 35 23
1.2 28 21
1.2 26 19
1.2 31 23
0.8 22 9
0.8 29 14
0.8 26 13
0.8 37 15
0.5 39 7
0.5 26 7
0.5 20 5
0.5 39 6
0 27 0
0 22 0
0 20 0
0 24 0
*Colo.Nick
1.5 23 20
1.5 28 26
1.5 33 29
1.5 27 25
1 31 24
1 24 18
1 24 17
1 35 22
0.66 27 12
0.66 24 10
0.66 29 14
0.66 23 11
0.44 21 6
0.44 25 4
0.44 28 7
0.44 26 5
0 25 0
0 24 0
0 28 0
0 29 0

=Rhizoglyphus robini

PoloPlus Version 2.0
Date: 05 OCT 2024
Rhizoglyphus robini
Data file: C:\Users\Samadieh\Desktop\Cholor+lead.txt
Number of preparations: 2
Number of dose groups: 32
Model: Probit
Natural Response Parameter: no
Convert doses to logarithms: yes
LDs: 50
~~~~~~

Rhizoglyphus robini

Intercepts and slopes unconstrained. Preparation is (1) Color
Not estimating natural response

	parameter	standard error	t ratio
Color	0.336	0.067	4.983
SLOPE	4.165	0.348	11.974

Variance-Covariance matrix

	Color	SLOPE
Color	0.454292E-02	0.543560E-02
SLOPE	0.543560E-02	0.120983

Chi-squared goodness of fit test

prep	dose	n	r	expected	residual	probab	std resid
Color	1.800	27.	26.	24.82	1.184	0.919	0.836
	1.800	24.	22.	22.06	-0.059	0.919	-0.044
	1.800	38.	36.	34.93	1.074	0.919	0.639
	1.800	34.	32.	31.25	0.750	0.919	0.472
	1.200	35.	23.	26.15	-3.152	0.747	-1.226
	1.200	28.	21.	20.92	0.079	0.747	0.034
	1.200	26.	19.	19.43	-0.427	0.747	-0.193
	1.200	31.	23.	23.16	-0.163	0.747	-0.067
	0.800	22.	9.	10.41	-1.406	0.473	-0.600
	0.800	29.	14.	13.72	0.283	0.473	0.105
	0.800	26.	13.	12.30	0.702	0.473	0.276
	0.800	37.	15.	17.50	-2.501	0.473	-0.823
	0.500	39.	7.	6.99	0.006	0.179	0.003
	0.500	26.	7.	4.66	2.337	0.179	1.195
	0.500	20.	5.	3.59	1.413	0.179	0.824
	0.500	39.	6.	6.99	-0.994	0.179	-0.415

chi-square: 6.281 degrees of freedom: 14 heterogeneity: 0.449

Effective Doses

		dose	limits	0.90	0.95	0.99
LD50	Color	0.831	lower	0.780	0.770	0.750
			upper	0.882	0.892	0.912

Intercepts and slopes unconstrained. Preparation is (2) Col.lead
Not estimating natural response

parameter standard error t ratio
~~~~~~

Col.lead	1.075	0.108	9.984
SLOPE	3.314	0.329	10.066

Variance-Covariance matrix

	Col.lead	SLOPE
Col.lead	0.115942E-01	0.271711E-01
SLOPE	0.271711E-01	0.108407

Chi-squared goodness of fit test

prep	dose	n	r	expected	residual	probab	std resid
Col.lead	1.200	23.	22.	20.92	1.082	0.909	0.786
	1.200	28.	26.	25.46	0.535	0.909	0.352
	1.200	30.	28.	27.28	0.716	0.909	0.456
	1.200	26.	25.	23.65	1.354	0.909	0.925
	0.800	27.	20.	20.91	-0.912	0.775	-0.420
	0.800	23.	18.	17.81	0.186	0.775	0.093
	0.800	28.	19.	21.69	-2.687	0.775	-1.215
	0.800	23.	17.	17.81	-0.814	0.775	-0.406
	0.500	28.	14.	14.86	-0.863	0.531	-0.327
	0.500	24.	12.	12.74	-0.740	0.531	-0.303
	0.500	21.	11.	11.15	-0.147	0.531	-0.064
	0.500	30.	14.	15.92	-1.924	0.531	-0.704
	0.300	25.	8.	6.38	1.618	0.255	0.742
	0.300	29.	7.	7.40	-0.403	0.255	-0.172
	0.300	24.	8.	6.13	1.873	0.255	0.877
	0.300	26.	7.	6.64	0.363	0.255	0.163

chi-square: 5.707 degrees of freedom: 14 heterogeneity: 0.408

Effective Doses

		dose	limits	0.90	0.95	0.99
LD50	Col.lead	0.474	lower	0.433	0.425	0.409
			upper	0.514	0.521	0.537

HYPOTHESIS OF EQUALITY (equal slopes, equal intercepts): REJECTED (P<0.05)
(chi-square: 71.41, degrees of freedom: 2, tail probability: 0.000)

HYPOTHESIS OF PARALLELISM (equal slopes): NOT REJECTED (P>0.05)
(chi-square: 3.16, degrees of freedom: 1, tail probability: 0.075)

Lethal dose ratio (LD50)

	ratio	limits	0.95
Col.lead	1.753	lower	1.546
		upper	1.988

=Rhizoglyphus robini

PoloPlus Version 2.0
Date: 05 OCT 2024
Rhizoglyphus robini
Data file: C:\Users\Samadieh\Desktop\Cholor+lead.txt
Number of preparations: 2
Number of dose groups: 32
Model: Probit
Natural Response Parameter: no
Convert doses to logarithms: yes
LDs: 50
~~~~~~

Rhizoglyphus robini
  Color   subjects 481   controls 93
    slope=4.165+-0.348   nat.resp.=0.000+-0.000
    heterogeneity=0.45
    LD50=0.831   95% limits: 0.770 to 0.892
  Col.lead   subjects 415   controls 95
    slope=3.314+-0.329   nat.resp.=0.000+-0.000
    heterogeneity=0.41
    LD50=0.474   95% limits: 0.425 to 0.521
  HYPOTHESIS OF EQUALITY: Rejected
  HYPOTHESIS OF PARALLELISM: Not Rejected
  LETHAL DOSE RATIOS
    LD50
      Col.lead ratio=1.753   95% limits: 1.546 to 1.988
~~~~~~
